# Integrative Proteomics Reveal Neuroimmune and Dopaminergic Alterations Across the Nociceptive Neuraxis in Neuropathic Pain

**DOI:** 10.3390/cells15030290

**Published:** 2026-02-04

**Authors:** Shreyasi Majumdar, Santosh Kumar Prajapati, Aishwarya Dande, Vinod Kumar Yata, Khushboo Choudhary, Ramalingam Peraman, Nitesh Kumar, Sairam Krishnamurthy

**Affiliations:** 1 Neurotherapeutics Laboratory, Department of Pharmaceutical Engineering and Technology, Indian Institute of Technology (Banaras Hindu University), Varanasi 221005, India; shreyasimajumdar.rs.phe18@itbhu.ac.in (S.M.); santosh.kumar.phe12@itbhu.ac.in (S.K.P.); 2 Department of Pharmaceutical Technology, School of Health and Medical Sciences, Adamas University, Kolkata 700126, India; 3 Department of Neurosurgery and Brain Repair, University of South Florida, Tampa, FL 33613, USA; 4 National Institute of Pharmaceutical Education and Research, Hajipur 844102, India; aishwarya01pa2021@niperhajipur.ac.in (A.D.); khushi11snehil@gmail.com (K.C.); drram@niperhajipur.ac.in (R.P.); nitesh.kumar04@niperhajipur.ac.in (N.K.); 5 Department of Pharmacology, School of Allied and Healthcare Sciences, Malla Reddy University, Hyderabad 500100, India; vinod.kumaryata@mallareddyuniversity.ac.in

**Keywords:** chronic constriction injury, neuropathic pain, neuroinflammation, orbitofrontal cortex, proteomics

## Abstract

Neuropathic pain (NP) arises from maladaptive changes in peripheral and central nociceptive circuits, yet molecular alterations spanning the entire pain neuraxis remain poorly understood. Neuroinflammation is increasingly recognized as a central mechanism in NP chronification, yet the region-specific molecular events linking immune activation to affective pain processing remain inadequately defined. In this study, we employed high-resolution LC-HRMS-based quantitative proteomics to investigate chronic constriction injury (CCI)-induced molecular alterations in the sciatic nerve (SN), spinal cord (SC), and orbitofrontal cortex (OFC) of male Wistar rats, a region critical for affective and cognitive pain modulation. Behavioral assessments confirmed the development of NP phenotypes and motor deficits. Proteomic profiling revealed exclusive and differentially expressed proteins enriched in neuroinflammatory pathways across all regions. S100 proteins (S100A8 and S100B) were significantly elevated in SN, SC, and OFC, as confirmed by immunofluorescence. Their up-regulation coincided with increased astrocyte (GFAP) and microglial (Iba-1) activation, highlighting a pervasive inflammatory milieu. Intriguingly, the OFC proteome demonstrated marked up-regulation of dopamine-regulating proteins and positive regulation of dopaminergic neurotransmission, suggesting involvement of reward-related analgesic circuits. Together, our findings delineate a “nociceptive neuraxis” driven by neuroimmune activation and neuromodulatory adaptations that interfaces with dopaminergic signaling to influence sensory and affective components of pain. This integrative molecular map highlights potential therapeutic targets, including glial-derived S100 proteins and dopamine modulators for the comprehensive management of NP.

## 1. Introduction

Neuropathic pain (NP) is a prominent clinical illness that arises as a consequence of injury or lesion of the primary afferent neurons of the somatosensory nervous system, resulting in a broad range of complex experiences that integrate sensory, emotional, and cognitive dimensions [1]. In the NP transmission pathway, the peripheral nervous system (PNS) detects and transmits nociceptive signals, while the central nervous system (CNS) plays a pivotal role in integrating, processing, and emotionally interpreting pain [2]. The global epidemiological studies suggested that NP affects around 7–10% of people worldwide [1,3] with greater rates documented in individuals with metabolic diseases (up to 26%) or in the case of spinal cord injury (around 40%) [4,5]. Despite over a century of study on nociception and analgesia, around 35% of NP patients do not respond to classical analgesics and even exhibit substantial side effects [6,7]. This is mostly owing to the fact that the molecular and cellular mechanisms contributing to NP are not entirely comprehended. Among the currently available treatments, opioids are considered highly efficacious in mitigating pain as they act at multiple levels of the nervous system [8,9]. Aside from this, their effectiveness stems from their ability to alleviate not only the sensory attributes of NP but also reduce its emotional aspect by acting on the limbic and reward circuits in the brain, particularly those involving the ventral tegmental area (VTA) and nucleus accumbens (NAc) [10]. This ability of opioids to attenuate both the sensory and emotional dimensions of NP underlies their perceived effectiveness and clinical preference. However, prolonged opioid use leads to tolerance and addiction [11], necessitating the need to elucidate the protein networks expressed in the nociceptive neuraxis connecting the PNS and CNS, which will assist in discovering novel molecular targets for the management of NP without the side effects that are commonly associated with opioids.

Peripheral nerve injury triggers nociceptive signaling that ascends through the spinal cord (SC) [12,13] to supraspinal regions, including the periaqueductal gray (PAG) and thalamus (TH), ultimately reaching cortical structures comprising the “pain neuromatrix”, such as the somatosensory cortex (SSC), prefrontal cortex (PFC), insula (INS), and anterior cingulate cortex (ACC) [14]. While the SSC primarily encodes the sensory-discriminative aspects of pain, the PFC, INS, and ACC govern affective, emotional, and interoceptive components [14,15]. Most of the research on algology has focused on the midbrain-mediated descending pathways (PAG and rostroventral medulla), which the classical analgesics and opioids commonly target [16,17]. Conversely, during chronic NP conditions, there are abnormalities in these “opioid-sensitive” top-down pain inhibitory circuitries leading to impaired conditioned pain modulation (CPM) [16,18], contributing to the persistence and severity of NP [19]. Notably, current evidence suggests a dissociation between sensory and modulatory networks in the brain, highlighting a lack of integrated understanding of pain processing [20,21]. Moreover, NP is a subjective and emotionally laden experience, and the majority of research emphasizes sensory pathways rather than the emotional dimension, essential for the patient’s pain perception. To address this, we propose a “nociceptive neuraxis” that encompasses sensory, emotional, and cognitive domains, aiming to identify novel molecular targets through neuroproteomics.

In the context of the nociceptive neuraxis, the orbitofrontal cortex (OFC) emerges as a critical hub for integrating the emotional and cognitive dimensions of pain [22]. Located in the prefrontal lobe, the OFC is extensively interconnected with brain regions responsible for emotional regulation (such as the amygdala and ACC), interception (INS), and reward processing (NAc), thus affecting the overall pain perception [23,24,25]. OFC is also connected to the hippocampus and thus controls memory formation related to chronic pain experiences [26]. Therefore, the OFC’s role in decision-making and executive functions adds cognitive control over pain, affecting perception and management. Moreover, OFC has strong connections with brain regions involved in descending pain inhibitory signals that reduce pain transmission at the spinal level [23,24]. Clinically, functional imaging studies have also demonstrated that OFC activity is inversely correlated with activation of classical pain-processing areas such as the ACC and INS during positive reinforcement scenarios, such as monetary gain, indicating its role in pain inhibition via reward pathways [25]. Furthermore, the Field’s motivation–decision model posits that pain is a dynamic perceptual experience shaped by competing behavioral priorities and motivational drives [27]. Hence, we propose OFC to be the central point of pain neural circuits because reward and other motivational states influence the perception of pain. Therefore, we chose the sciatic nerve (SN), SC, and OFC as a pain-mediating circuit so that we can decode different proteins involved in the pathogenesis and neurobiology of NP for newer therapeutic interventions.

Animal models are essential for studying disease pathophysiology because they mirror clinical symptoms and molecular and neurophysiological changes in patients [28]. One such peripheral nerve injury model for NP is the chronic constriction injury (CCI) where loose ligatures are tied around the common SN [29]. The CCI model holds clinical relevance and presents face validity as the animal exhibits similar painful and abnormal peripheral neuropathies, as observed clinically [29]. Further, in terms of predictive validity, the CCI model is widely preferred for drug discovery as the pain symptoms persist for a longer time [30]. Furthermore, in the CCI model, previous studies have mostly explored the neural connections between the peripheral nerve and spinal and supraspinal regions up to the subcortical regions of the brain [31,32]. However, there is a lack of information on the proposed pain neuraxis in this widely used NP rodent model. Hence, in this study, we have chosen the CCI animal model for a better understanding of the neurobiology of NP, identifying newer therapeutic targets in the proposed pain axis using neuroproteomics for the translation of preclinical findings to clinical practice.

Therefore, in our observational study, NP was induced by CCI of the SN in rats, and the establishment of NP symptoms was confirmed by various behavioral tests. The changes in the expression of differentially expressed proteins (DEPs) in the pain axis, comprising SN, SC, and OFC were elucidated using LC-HRMS. Following this, the Gene Ontology (GO) function, Kyoto encyclopedia of genes and genomes (KEGG) pathways, and functional analysis were performed for the DEPs between the control and CCI-induced NP rats in order to obtain a deeper understanding of the possible pathophysiology at the protein level from our findings. Further, the bioinformatics and functional annotation were also performed for the proteins exclusively expressed in the CCI rats, and the comparative analysis of the canonical pathways between the SN, SC, and OFC was evaluated. This study further investigated alterations in key neuroinflammatory markers, including TNF-α and IL-6, along the proposed pain neuraxis, alongside quantification of dopamine levels. In addition, changes in the expression of glial activation markers-S100 proteins (S100B and S100A8), GFAP, and Iba-1 were assessed through immunofluorescence analysis.

## 2. Materials and Methods

### 2.1. Materials

The RIPA buffer was purchased from Himedia (Dindori, India) and the protease inhibitor cocktail was obtained from SRL (Taloja, India). The BCA Protein Assay kit and trypsin were procured from Merck (St. Louis, MO, USA) and Thermo Fisher Scientific (Rockford, IL, USA), respectively. Dithiothreitol (DTT) and iodoacetamide (IAA) were purchased from G Biosciences. Water and acetonitrile (ACN) are of MS grade and were of JT Baker with a purity > 99.9%. Ammonium bicarbonate and other chemicals used in the study were obtained from Sigma-Aldrich (St. Louis, MO, USA).

### 2.2. Animals

Adult male Wistar albino rats of weight 200 ± 20 g were used in the experiment to avoid estrogen-mediated modulation of pain perception. The rats were procured from the Central animal house, IMS-BHU, Varanasi, India. Before starting the experiment, the rats were acclimatized for a week at an ambient and controlled temperature of 25 ± 1 °C with a 12-h light and dark cycle. The experimental animals had ad libitum access to a standard laboratory diet and water throughout the experiment. The rats were housed in cages (430 × 270 × 150 mm) with corn cob bedding. All efforts were made to minimize the number of animals used during the experiment. The studies were performed in accordance with the guidelines of the Committee for the Control and Supervision of Experiments on Animals (CCSEA), Government of India and “Care and Use of Experimental Animals” (Vol. 1, second ed., 1993, and Vol. 2, 1984) guidelines of the National Institute of Health, U.S.A. The experimental protocol for the present study was approved by the Institutional Animal Ethical Committee (Ref No. IIT(BHU)/IAEC/2023/II/085 and IIT(BHU)/IAEC/2024/I/022).

### 2.3. Animal Experimental Design for the Proteomics Study

**Figure 1:**
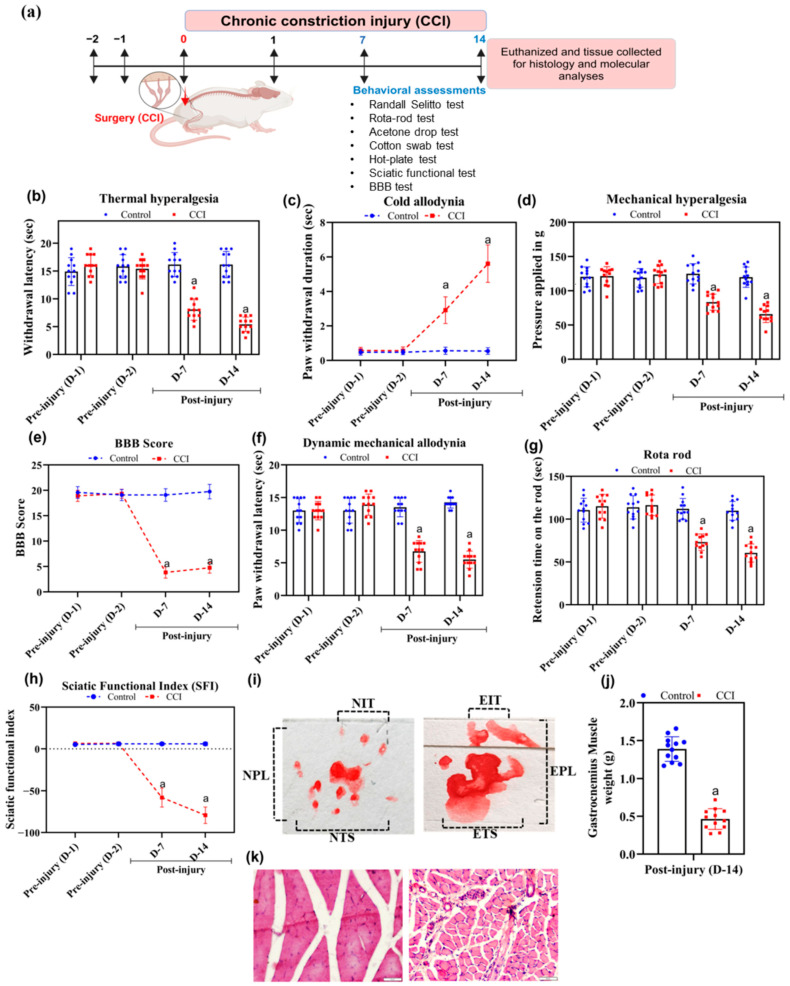
(**a**) Schematic representation of the experimental protocol followed for the CCI-induced neuropathic pain model and the behavioral assessment of the symptoms developed post-surgery. Effect of CCI on (**b**) thermal hyperalgesia, (**c**) cold allodynia, (**d**) mechanical hyperalgesia, (**e**) BBB score, (**f**) dynamic mechanical allodynia, and (**g**) retention time on the Rota-rod on day 7 and 14 post-surgery. (**h**) Assessment of the effect of CCI on the locomotor function by walking track analysis. (**i**) Image representation of footprint for calculating SFI. (**j**) Effect of CCI on the gastrocnemius muscle weight and (**k**) representative image of the histological analyses of gastrocnemius muscle stained with hematoxylin and eosin at the end of 14th day post-surgery. All values are in mean ± SD (*n* = 12 rats/group). NPL: normal print length; EPL: experimental print length; NIT: distance between intermediary toes of normal foot; EIT: distance between intermediary toes of experimental (injured) foot; NTS: total spreading of toes of normal foot; ETS: total spreading of toes of experimental (injured) foot. ^a^ *p* < 0.05 compared to control. (The gastrocnemius muscle weight was analyzed by Student’s *t*-test, and two-way ANOVA followed by the Bonferroni post-hoc test were followed for the other behavioral tests).

G*power analysis was performed before devising the experimental protocol to calculate sample size within the type I and II error limit. We used 0.05 as α error probability and 0.8 as power. The effect size was 0.25 as stated by Cohen [33] and the non-sphericity correction (€) was 1. In this investigation, there are two groups, and the total sample size was estimated at 22 utilizing a priori power analysis. Therefore, we had taken twelve male Wistar rats per group (*n* = 12 rats/group). All the male Wistar rats were weighed and were randomly divided into two groups, i.e., naïve control and peripheral-nerve-injured (CCI) group using the randomization technique. Naïve controls were chosen to establish an actual basal proteomic reference state in the proposed nociceptive neuraxis, facilitating the precise identification of CCI-induced molecular reprogramming without interference from inflammatory responses related to incision or suturing. NP was induced by CCI and behavioral assessments (hot-plate test, Randall Selitto test, cotton swab test, acetone drop test, Rota rod test, sciatic functional index (SFI), BBB (Basso, Beattie, and Bresnahan) test) were performed before the surgery and on day 7 and 14 post-surgery to access pain along with the motor and sensory deficits. Additionally, the body weight of the experimental animals and their food and water intake were monitored throughout the experiment. On day 14, the anaesthetized animals were sacrificed by decapitation (3% *v*/*v* isoflurane inhalation; R620 veterinary anesthesia machine, RWD Life Science, San Diego, CA, USA). Day 14 post-CCI was selected as the endpoint as this time point represents the established severity of chronic NP phase, during which hyperalgesia/allodynia and neuroimmune changes are fully stabilized [34]. The SN of the ipsilateral side of the leg, SC, and OFC were dissected and the tissues were processed for proteomics study (Figure 1a).

### 2.4. Chronic Constriction Injury (CCI) Model of Neuropathic Pain

The NP was induced in the rats by CCI of the sciatic nerve [34]. The experimental animals were anaesthetized intraperitoneally using ketamine (80 mg/kg) and xylazine (10 mg/kg). Once the surgical level of anesthesia was attained, the rats were placed on the thermo-regulated heating mat maintained at 37 °C. Then the skin of the right leg was shaved and sterilized and an incision parallel to the femur was made. The sciatic nerve (SN) was exposed by gluteal muscle incision. Around 10 mm of the SN proximal to the trifurcation was freed from the surrounding connective tissue and four loose ligatures (silk 4/0; Ethicon, Raritan, NJ, USA) were tied with a gap of 1 mm around the nerve. The constriction of the SN while tying the ligatures was performed until a brief twitch was observed to prevent obstruction of the epineural blood flow. Then, the muscle was sutured using 6/0 reabsorbable suture and the animals were kept under post-operative care. In order to limit the local infection, iodine solution was applied prior to and after the suturing of the incision.

### 2.5. Behavioral Analysis

The experimental animals were acclimatized to the testing environment for at least two days before the baseline behavioral testing. The behavioral assessments were performed on days 7 and 14 post-surgery (Figure 1a). An investigator blinded to treatment groups conducted behavioral testing, SFI assessment, and histological analysis.

#### 2.5.1. Thermal Hyperalgesia (Hot-Plate Test)

The development of heat hyperalgesia in rats post-surgery was assessed using Eddy’s hot plate (Orchid Scientific, Nashik, India) [35]. The surface of the plate was preheated and the temperature was maintained at 52.5 ± 0.5 °C. Then, animals were positioned on the heated plate and the nocifensive withdrawal reflex latency, such as the uplifting and licking of the rear paw of the injured side, was recorded as the nociceptive threshold. The cut-off time of 20 s was maintained to avoid burn injury to the animals.

#### 2.5.2. Cold Allodynia (Acetone Drop Test)

The development of pain sensation due to non-noxious stimuli (cold) post-CCI surgery was evaluated by the acetone drop test. In this test, rats were first acclimatized in a chamber with a mesh floor for 20 min and then 100 µL of acetone was dropped on the dorsal surface of the hind paw of the injured leg. Cold allodynia produced in response to the evaporation of acetone was indicated by the repeated withdrawal, licking, biting, or flinching of the paw. The total duration of withdrawal of the paw in air until it was placed back on the mess surface was recorded in seconds [35].

#### 2.5.3. Mechanical Hyperalgesia (Randall Selitto Test)

Hyperalgesia to the mechanical stimulation was measured in terms of the response mechanical nociceptive threshold to the pressure applied on the paw of animals using the digital paw pressure analgesiometer (Orchid Scientific, Nashik, India) based on the Randall Selitto test [36,37]. In this test, an increasing mechanical force was applied focally to the dorsal surface of the hind paw of the injured leg using the pressure applicator until a withdrawal nociceptive response was observed. The pressure at which the rats vocalized or displayed a painful response (flinching or withdrawal reaction of the paw or leg upon stimulation) was taken as the endpoint and recorded. The nociceptive threshold was defined as the mean of three subsequent readings, converted to force in grams.

#### 2.5.4. Dynamic Mechanical Allodynia (Cotton Swab Test)

The dynamic mechanical allodynia developed after the CCI-induced NP were detected by the cotton swab test in rats [38]. The animals were first habituated in the plexiglass chamber with the mesh flooring before the test session in order to minimize their normal movements during the test period. Then, using a cotton swab, the plantar part of the ipsilateral hind paw of rats was stroked gently and the latency of paw withdrawal was recorded with a cut-off time of 15 s.

#### 2.5.5. Rota Rod

The rotarod test has been employed for assessing rats’ motor coordination capability post-surgery [39]. The animals being studied are trained for two consecutive days on the Rota rod (IKON instruments, Delhi, India) at the lowest speed (i.e., 5 rpm) so that they attain stable baseline performances. Then, during the test session, the speed of the rotating rod was increased to 15 rpm. The total time spent by the animals on the rod was recorded with a maximum cut-off time of 300 s.

#### 2.5.6. Sciatic Functional Index (SFI)

SFI was calculated using the walking track analysis in which the animals were made to walk on a straight track (8.3 × 43 cm) that was darkened at one end [40,41]. The SFI value ranges from 0 to −100, with SFI value 0 meaning normal while −100 denotes total impairment. After two or three trials on the track, the rats were conditioned so that they walked directly to the darkened end without exploring the track. Then a piece of white paper was placed on the walking track and the hind paws of the animals were dipped in different color solutions (the normal left paw with green and the injured right paw with red). The animal was then placed at the entrance of the track and was allowed to walk over the paper. The marks on the paper were dried, and the following measurements were made:

##### Print Length (PL): The Length of One Footprint of Normal (NPL) and Experimental Side (EPL)

Total spreading (TS): The linear distance between the center of the first toe print and the center of the fifth toe print of normal (NTS) and experimental side (ETS).

Distance between intermediary toes (IT): The linear distance between the center of the second toe print and the center of the fourth toe print of normal (NIT) and experimental side (EIT).

The SFI was calculated using the following formula:

SFI = −38.8(EPL-NPL)/NPL + 109.5(ETS-NTS)/NTS + 13.3(EIT-NIT)/NIT − 8.8

#### 2.5.7. BBB (Basso, Beattie, and Bresnahan) Locomotor Test

The BBB test is normally performed to assess the deterioration of the motor function post-CCI of the SN in rats [42]. A score of 0 to 21 was given according to the movement of the animal by a trained observer who was blinded to the experiment. A score of 0 was awarded when there was no spontaneous movement while the rats scored 21 when they exhibited normal limb movement. Normally a score of 14 was given to an animal that showed complete limb coordination. In this experiment, the animals under study were placed in an open circular enclosure, observed for a period of 4 min and scored accordingly.

### 2.6. Gastrocnemius Muscle Mass Assessment

The injury of the sciatic nerve leads to atrophy of the largest muscle supplied by it, i.e., the gastrocnemius muscle, which was assessed using the weight ratio of the gastrocnemius muscle 14 days after the surgery [43]. After the animals are euthanized, the gastrocnemius muscle was dissected from the contralateral and ipsilateral sides and weighed when still wet using an electronic balance.

### 2.7. Histological Analysis

The gastrocnemius muscle was fixed in 10% buffered formalin solution, which was sliced (5 µm) using the cryostat. Then, the finely sliced sections were stained with hematoxylin followed by counterstaining with eosin. Then, the tissue sections were dehydrated with a graded series of alcohol and mounted with dibutyl phthalate xylene (DPX). The slides were then observed under the microscope (Olympus, Japan) for any structural abnormalities [44].

### 2.8. Sample Preparation for Proteome Analysis

#### 2.8.1. Protein Extraction and Quantification

The OFC, SN, and SC were weighed separately without pooling the samples and were homogenized in RIPA (radioimmunoprecipitation assay) buffer under ice-cold conditions. Then the homogenate was thoroughly mixed with a complete protease inhibitor cocktail and kept on ice for 30 min. The above mixture was centrifuged at 10,000 rpm at 4 °C for 20 min and the supernatant was collected into fresh Eppendorf tubes. The protein concentration was estimated using the BCA (bicinchoninic acid) assay method [45]. A total of 150–200 µg/mL extracted protein from OFC, SC, and SN was precipitated overnight by mixing four times the volume of precooled acetone and incubating the mixture at −20 °C overnight. Subsequently, the extracted protein was pelleted by centrifuging at 15,000 *g* for 10 min at 4 °C. The protein pellet was air dried gently to eliminate excess acetone.

#### 2.8.2. In-Solution Trypsin Digestion

The protein pellets were reconstituted in 7 M Urea and 50 mM ammonium bicarbonate solution (pH 7.5 to 8.0) for denaturation for 10 min. Then, the protein samples were reduced at 37 ± 2 °C in the presence of 200 mM dithiothreitol (in 50 mM ammonium bicarbonate solution) for 1 h. The above mixture was alkylated in the dark for 1 h in 200 mM iodoacetamide (in 50 mM ammonium bicarbonate solution). To consume the unreacted iodoacetamide, 200 mM DTT was added and incubated at room temperature in the dark. Further, 1 mM calcium chloride (in 50 mM ammonium bicarbonate) was added to lower the urea concentration to 0.5 M. Enzymatic digestion was initiated by adding trypsin in a final ratio of 1:50 (*w*/*w*, trypsin:protein) and incubating the mixture for 16 h at 37 °C. Later on, to inhibit the enzyme activity, the peptide sample was acidified with formic acid. The digested samples were dried using a vacuum concentrator (Thermo Fisher Scientific, Rockford, IL, USA). The purified peptides were then collected and stored at −80 °C until further analysis.

#### 2.8.3. Sample Cleanup/Desalting

The sample solution obtained by trypsin digestion contains not only peptides but also other compounds, such as salts, which might contaminate the MS equipment and reduce its sensitivity. Prior to LC-HRMS injection, samples were cleaned with desalting tips to avoid sample clogging in the column and flow channel, especially in NanoLC, while keeping the MS device clean and concentrating peptide samples. The protein samples are desaltED USING A C18 SPIN COLUMn (Thermo Fisher Scientific). The desalted samples were then dried in a speed vacuum (Thermo Fisher Scientific) and could be saved for later use or injected (1 µg) in the Nano LC by reconstituting the samples with 40 µL 100% water and 0.1% formic acid.

#### 2.8.4. Measurement Conditions for the LC-HRMS

Nanoscale LC separation of peptides was performed with a Rapid Separation Liquid Chromatography (RSLC) Nano system (Thermoscientific Ultimate Dionex 3000), equipped with an RSLC pump (Loading and Nano pumps), RS autosampler, trap/loading column (ThermoFisher Scientific Acclaim PepMap 100 (75 µm × 2 cm, C18, 3 µm 100 Å)), and analytical reverse-phase column (ThermoFisher Scientific PepMap RSLC C18, 75 µm × 25 cm, 2 µm, 100 Å). Peptide analysis was carried out using method parameters similar to the previously described method, with slight modifications. The mobile phase A consisted of 0.1% formic acid in water while mobile phase B contained 0.1% formic acid in ACN for the nano pump. Similarly, 0.1% formic acid in water was used for the loading pump. The peptides were separated with a gradient of 0 to 20 min, 5% B; 21–60 min, 50% B; 61–130 min, 95% B; 121–130 min, 5% B at a flow rate of 0.3 µL/min. The column and MS were re-equilibrated at initial conditions for 20 min. The column temperature was maintained at 40 °C and the autosampler temperature was maintained at 5 °C. The samples were initially transferred with 100% water, 0.1% formic acid solution to a loading pump with a flow rate of 5 µL/min for 4 min, and the injection volume was 2 µL. Further, MS parameters were as follows: positive spray voltage was maintained at 1900 V, negative spray voltage was at 600 V, ion transfer tube temperature was at 290 °C, Automatic Gain Control (AGC) target was set at 300% with an IT of 25 MS. A full MS scan was acquired from 350 to 2000 *m*/*z* in the Orbitrap at a resolution of 60,000, followed by the MS^2^ scans at a resolution of 15,000 in a data-dependent acquisition (DDA) model (20 scans). The dynamic exclusion window was at 20 s, the intensity threshold at 5.0 e^3^, and the charge state was set to 2–6. The 20 most intense peaks from the survey scans were chosen for fragmentation using higher-energy collisional dissociation (HCD) with a normalized collision energy of 30%, target intensity values of 5000 charged, resolution of 15,000, and a maximum IT of 50 ms.

### 2.9. Proteomics Data Analysis

The LC-MS/MS raw datasets generated were uploaded into the Proteome Discoverer, version 2.5 (Thermo Fischer, San Jose, CA, USA) and were searched for Label Free Quantification (LFQ) analysis from the UniprotProteomes protein database (UP000002494 *Rattus norvegicus*; 13 September 2023, 83,190 sequences). A total of eighteen LC-MS raw data files were organized into three groups, each representing a unique combination of sample descriptions as control and CCI. The software utilized the sample files in two distinct processes: protein identification during the processing step and LFQ during the consensus step. In the LFQ dataset, eight groups were formed, each with three biological repeats.

In a nutshell, the process of protein identification (sample processing) involved six processing nodes in PDv.2.5. This began with a spectrum file recalibration node, followed by a Minora Feature Detector and a Precursor Detector node. These steps were crucial for interpreting chimeric MS/MS spectra. We established a static modification for N-terminal acetylation and carbamidomethylation of cysteine. Additionally, a dynamic modification was set for methionine (oxidation), and a minimum missed cleavage number of two was designated. Subsequently, SEQUEST nodes, in conjunction with percolator, executed protein identification for the sample’s raw MSMS data, encompassing peptides with various common amino acid modifications.

In the LFQ consensus step, 15 PDv.2.5 nodes were involved. These nodes encompassed data quality threshold settings, Feature Mapper for chromatographic alignment, and Precursor Ion Quantifier for LFQ quantitation parameters. Importantly, the Precursor Ion Quantifier node included parameters for protein abundance calculation, protein ratio calculation using the median protein abundance of three biological repeats, and an individual protein intensity-based *t*-test for *p*-values and adjusted *p*-values assigned to protein ratios. The Peptide and Protein Filter node allowed only high-quality results, requiring at least two peptide sequences for protein identification (protein FDR confidence set to “high”). Protein annotation utilized the “*Rattus norvegicus*” database. The Protein Scorer calculated scores based on identified PSMs, and the Protein FDR Validator assigned confidence based on experimental *q*-values derived from FDRs at various score thresholds.

### 2.10. Bioinformatics Analysis

#### 2.10.1. Identification of Differentially Expressed Proteins (DEPs)

At first, the abundance ratio (log_2_Fold Change and *p*-value) (CCI/Control) and the accession of the proteins expressed in OFC, SC, and SN were collected from the datasets generated from Proteome Discoverer. Then DEPs expressed in both groups were identified and displayed using the Venn diagram. VENNY version 2.1 was used to present the overlapping proteins between the CCI and control group (https://bioinfogp.cnb.csic.es/tools/venny/index.html; accessed on 13 September 2023) [46]. Further, the up- and down-regulated proteins were filtered using two criteria: −1 > log_2_FC > +1 and *p*-value < 0.05 and were represented in a volcano plot.

#### 2.10.2. Functional and Pathway Enrichment Analysis

Then Gene Ontology (GO) mapping and Kyoto Encyclopedia of Genes and Genomes (KEGG) pathway analysis of the up- and down-regulated proteins in OFC, SC, and SN were performed using the DAVID Bioinformatics Resources version 6.8 (https://davidbioinformatics.nih.gov/summary_new.jsp; accessed on 13 September 2023) with the threshold *p*-value < 0.05 [47]. The GO comprises biological process (BP), cellular component (CC), and molecular function (MF) terms. Similarly, the exclusive proteins expressed in the CCI group are considered to be involved either in the progression of disease or in the repair mechanisms post-injury. Thus, the GO analysis was also performed for the CCI-exclusive proteins. The bubble dot-plot diagram was used for the graphical display using the free online platform (http://www.bioinformatics.com.cn/; accessed on 14 September 2023).

### 2.11. Ingenuity Pathway Analysis (IPA)

The pathway topology software, IPA^®^ (QIAGEN; https://www.qiagenbioinformatics.com/products; accessed on 15 September 2023) was used to elucidate the biological significance of the DEPs expressed in the OFC, SC, and SN by analyzing the canonical pathways from the QKB (QIAGEN Knowledge Base). IPA identifies networks of interacting proteins and connects these proteins in the dataset to molecular networks available within the Ingenuity Knowledge Database. The identified proteins were analyzed using IPA Core analysis to assess functional pathways in the dataset. Right-tailed Fisher’s exact test was used to calculate the *p*-value for determining the significance of each canonical pathway, and *p*-values < 0.05 were considered statistically significant. A *Z* score ≥ 2 or ≤ −2 in the canonical pathway was considered significant activation or inhibition, respectively. Further, the upstream regulator and molecular networks analysis were also determined.

### 2.12. Immunofluorescence

SN, SC, and OFC were fixed in 4% paraformaldehyde for 24 h followed by the antigen retrieval protocol. The tissue sections were then cut into 5-µm-thick sections on the slides coated with 2% APES (3-aminopropyl triethoxysilane) using a cryostat microtome (Leica, Teaneck, NJ, USA). Following this, the tissue sections were permeabilized in 0.2% Triton X-100 and blocking was performed for 1 h. The sections were then incubated in one or another of the primary antibodies (anti-GFAP antibody (No.: ab7260, Abcam, Waltham, MA, USA) or anti-S100b antibody (No. E-AB-60087; Elabscience Biotechnology Co., Ltd., Houston, TX, USA) or anti-S100A8 antibody (No. A1688; ABclonal, Wuhan, China) or anti-Iba1 antibody (No. 019-19741; FUJIFILM Wako Chemicals, Richmond, VA, USA)). After washing three times in PBS, a secondary antibody goat anti-rabbit IgG FITC (ab6717; Abcam) or goat anti-rabbit IgG TRITC (ab6718, Abcam, USA) incubation was performed and counterstained with 1 µg/mL of 4′6-diamidino-2-phenylindole (DAPI). The slides were mounted in DABCO (Sigma, St. Louis, MO, USA), then sealed and observed under a fluorescence microscope (Olympus BX53). Images captured were analyzed using Image J (NIH, Bethesda, MD, USA) [48].

### 2.13. Assessment of TNF-α, IL-6, and Dopamine (DA) Protein Levels in the Proposed Neuraxis

TNF-α (No: KB3145, Krishgen Biosystems, Mumbai, India), IL-6 (No: KB3068, Krishgen Biosystems, India), and dopamine (No: KLR0219, Krishgen Biosystems, India) ELISA Kits were used in this study to quantitatively assess the protein levels in SN, SC, and OFC following the manufacturer’s instructions. The protein concentrations were determined using the Bradford method [49] and the findings were subsequently reported as the concentration of dopamine and proinflammatory cytokines in ng/mg protein and pg/mg protein, respectively.

### 2.14. Statistical Analysis

All data are represented as Mean ± SD and the statistical analysis was performed using the GraphPad Prism 8. The behavioral tests such as hot-plate test, acetone drop test, Randall Selitto test, cotton swab test, Rota rod test, BBB score, and SFI were analyzed statistically using the two-way ANOVA followed by Bonferroni post-hoc test. The gastrocnemius muscle weight and the changes in the proinflammatory cytokines (TNF-α and IL-6) and dopamine level were analyzed by Student’s *t*-test. Similarly, for the immunofluorescence expression of proteins (GFAP, Iba-1, S100B, and S100A8), Student’s *t*-test was followed. In all the tests, differences were considered statistically significant when the *p*-value was <0.05 in the overall data analysis.

## 3. Results

### 3.1. Effect of CCI on the Sensory and Motor Functions in Rats

The behavioral tests were performed on days 7 and 14 post-CCI surgery to ascertain the development of NP in rats (Figure 1a). The CCI animal model has been reported to engender hyperalgesia and allodynia in response to various stimuli along with muscular atrophy of the lower leg [50]. Similarly, in our study, statistical analysis by two-way ANOVA followed by the post-hoc test revealed the development of thermal hyperalgesia, cold allodynia, mechanical hyperalgesia, and dynamic mechanical allodynia (*p* < 0.05) compared to the control rats from day 7 onwards (as shown in Figure 1b–f). Peripheral nerve injury caused significant reduction in the withdrawal latency when placed on the hot plate (Figure 1b) along with the elevation in paw withdrawal duration on dropping acetone on the injured paw (Figure 1c) among the groups ([F(_1,88_) = 117.4; *p* < 0.05] and [F(_1,88_) = 351.4; *p* < 0.05], respectively), time ([F(_3,88_) = 33.98; *p* < 0.05] and [F(_3,88_) = 114.3; *p* < 0.05], respectively), and their interaction ([F(_3,88_) = 48.21; *p* < 0.05] and [F(_3,88_) = 135.8; *p* < 0.05], respectively). Further, it was also observed that the threshold to withstand pressure applied on the dorsal part of the paw decreased significantly during the Randall Selitto test (Figure 1d) along with statistically lower BBB score among the group ([F(_1,88_) = 1084; *p* < 0.05]), time ([F(_3,88_) = 336.4; *p* < 0.05]), and their interaction ([F(_3,88_) = 342.7; *p* < 0.05]) as shown in Figure 1e. Post-CCI of SN, there was development of dynamic mechanical allodynia (Figure 1f) and the rats also developed motor in-coordination and decreased muscle strength as indicated by the lesser time spent by the rats on the Rota-rod (Figure 1g) across the group [F(_1,88_) = 66.93; *p* < 0.05], time [F(_3,88_) = 35.52; *p* < 0.05], and their interaction [F(_3,88_) = 31.31; *p* < 0.05]. Additionally, the SFI of the experimental rats on days 7 and 14 reduced and was found to be −58.08 ± 11.34 and −79.25 ± 9.87, respectively compared to 6.58 ± 1.31 before surgery, suggesting the development of motor incoordination (Figure 1h,i).

Moreover, post-CCI injury, there was a significant reduction in the weight of the gastrocnemius muscle of the ipsilateral side of CCI rats (i.e., 0.46 ± 0.136 g) compared to the control rats, i.e., 1.39 ± 0.161 g, suggesting muscle atrophy (Figure 1j). Similarly, the histological analyses of the gastrocnemius muscle of control rats revealed homogeneous morphology with the densely packed polygonal shaped skeletal muscle fibres along with the peripheral oval nuclei. On the other hand, the CCI group exhibited fragmentation of the sarcoplasm with mononuclear cell infiltration with the widening of endomysium observed in injured rats (Figure 1k).

### 3.2. Quantification of Differentially Expressed Proteins (Deps) and Identification of Up- and Down-Regulated Proteins in the Sciatic Nerve

To elucidate the molecular alterations in SN following peripheral nerve injury, we performed high-resolution mass spectrometry-based proteomic analysis in control and CCI-induced NPrats.The proteome profiling identified the expression of 3994 and 4388 proteins in the SN of the control and CCI group, respectively. Further, a total of 3875 DEPs, representing 86%, were identified in SN when comparing the CCI group to the control, as depicted in the Venn diagram shown in Figure S1a. We also observed that 513 (11.4%) proteins were exclusively expressed in the CCI rats, while 119 (2.6%) proteins were exclusive to the control group. Furthermore, within the DEPs, the proteins exhibiting significant up-regulation and down-regulation in the SN were identified and illustrated in the volcano plot (Figure S1b). A total of 91 proteins were significantly up-regulated, whereas 168 proteins were significantly down-regulated in the SN on day 14 following the CCI (Figure S2).

The top 10 up-regulated proteins in the SN post-CCI were S100 calcium-binding protein A8 (Calgranulin A; A6J6Q3), a RIKEN cDNA-like protein (A6JIC0), zinc finger protein 551 (A0A8I5Y616), translocon-associated protein subunit gamma (F1M7T6), defensin NP-4 (A6IW99), NPC intracellular cholesterol transporter 1 (A0A8I6GB30), protein phosphatase 1G (F1LNI5), ribosomal protein S6 kinase (A6HZI4), Histone H3 (D3ZJ08), and cathepsin D (Q6P6T6) (as shown in Table 1). Similarly, the top 10 proteins among the 168 statistically significantly down-regulated proteins were non-specific serine/threonine protein kinase (A0A0G2JUP3), LON peptidase N-terminal domain and ring finger 1 (A0A0G2K313), albumin, isoform CRA_b (A6KKG1), myelin basic protein (MBP; A6K5J1), Chymase 1 (A6KH67), nuclear pore membrane glycoprotein 210 (A6IB90), zinc finger protein 831 (A0A0G2JZX4), keratin 83 (A7M746), cysteine and glycine-rich protein 1 (A6ICG2), and RCG61079 isoform CRA_a (A6JKY8) (Table 1).

### 3.3. Functional Annotation of Up- and Down-Regulated Proteins Expressed in the Sciatic Nerve Post-CCI Injury

**Figure 2:**
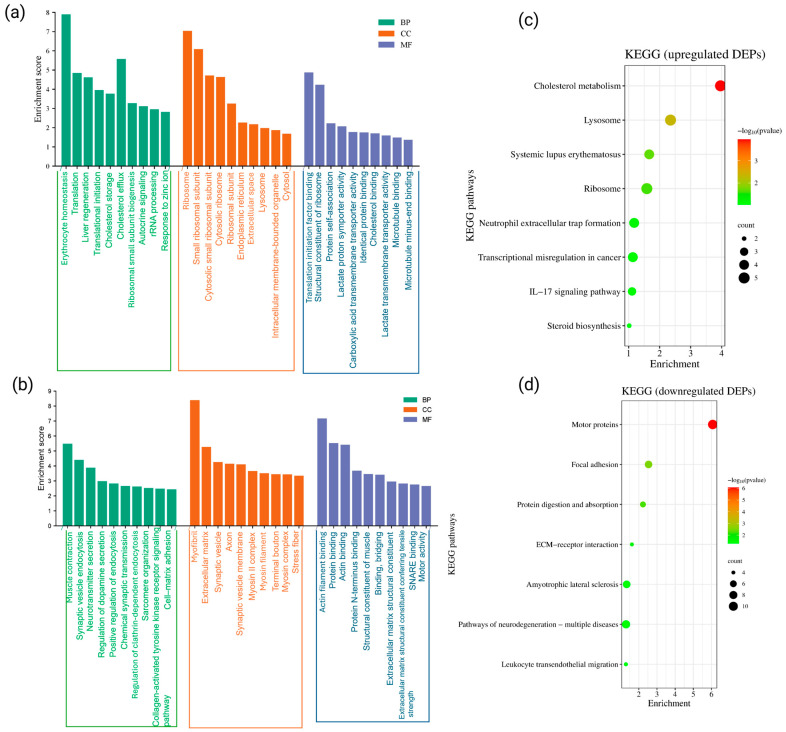
Gene ontology and KEGG pathway analysis of the up-regulated and down-regulated proteins in the sciatic nerve post-CCI. The bar plot representing the gene ontology annotation of (**a**) up-regulated and (**b**) down-regulated proteins in SN of rats post-injury. KEGG pathways analysis enriched by (**c**) up-regulated and (**d**) down-regulated DEPs are represented using the dot plot. The vertical axis represents the KEGG pathways and the horizontal axis represents enrichment, i.e., −log_10_ (*p*-value) of the associated pathway. The circles denote the number of proteins involved in a particular pathway.

To comprehensively evaluate the functional implications of DEPs in the SN following CCI, GO analysis of the up-regulated and down-regulated proteins was performed using the DAVID Bioinformatics Resources, which are represented in Figure 2a,b. Among the up-regulated DEPs, the GO annotation coverage was high, with 94.3% mapped to BP, 93.1% to CC, and 89.7% to MF. In terms of significantly enriched BP, the up-regulated proteins in SN are mostly associated with erythrocyte homeostasis, translation of proteins, liver regeneration, cholesterol storage and efflux, ribosomal small subunit biogenesis, autocrine signaling, rRNA processing, and response to zinc ion (Figure 2a). These processes suggest heightened metabolic activity, ribosomal biogenesis, and homeostatic compensation in the injured SN microenvironment.

At the cellular component level, up-regulated proteins were predominantly associated with intracellular organelles such as the ribosome, endoplasmic reticulum (ER), lysosome, and intracellular membrane-bounded organelles. Further, in terms of MF, the up-regulated proteins were enriched in categories such as translation initiation factor binding, protein self-association, lactate: proton symporter activity, carboxylic acid transmembrane transporter activity, cholesterol binding, lactate transmembrane transporter activity, and microtubule binding. The enrichment of cholesterol-binding proteins is particularly relevant, as increased formation of cholesterol-rich lipid rafts (also known as inflammarafts) has been linked to neuroinflammation in both preclinical and clinical models [51,52]. Therefore, in our datasets, the up-regulated proteins in SN appear to contribute to neuroinflammatory signaling and plasticity-driven pain chronification.

Similarly, for the down-regulated DEPs, the GO coverage for down-regulated proteins was also substantial: 89.0% for BP, 96.6% for CC, and 82.2% for MF. Among the significantly enriched BP terms, muscle contraction, synaptic vesicle endocytosis, neurotransmitter secretion, regulation of dopamine secretion, positive regulation of endocytosis, chemical synaptic transmission, clathrin-mediated endocytosis, sarcomere organization, collagen-activated tyrosine kinase receptor signaling, and cell–matrix adhesion were significantly enriched in the SN (Figure 2b). Additionally, MF analysis revealed that down-regulated proteins were enriched in functional annotations such as protein binding and bridging, actin filament binding, structural constituents of muscle, extracellular matrix components conferring tensile strength, and motor activity (*p*-value for all the GO terms was <0.05). Apart from these, the proteins involved in SNARE binding were down-regulated as per the GO analysis.

Further, to determine the potential physiological pathways of the statistically up- and down-regulated DEPs in the SN post-CCI, KEGG pathway analysis was also performed and illustrated in Figure 2c,d, respectively. According to our analysis, 55.2% of the up-regulated DEPs and 45.9% of the down-regulated DEPs were annotated with known KEGG pathways. Among the up-regulated proteins, the most significantly enriched KEGG pathways included cholesterol and steroid biosynthesis and metabolism, inflammatory signaling (e.g., IL-17 signaling), neutrophil infiltration, lysosomal activity, and ribosomal pathways (Figure 2c). These pathways suggest elevated immune response and enhanced protein turnover in the injured nerve. Conversely, the KEGG analysis of down-regulated DEPs indicated significant enrichment in pathways such as focal adhesion and extracellular matrix (ECM)-receptor interactions (Figure 2d). These proteins are linked to structural integrity, cell–matrix interactions, and cytoskeletal support systems. In addition, several down-regulated proteins were also associated with neurodegenerative disease pathways and leukocyte migration.

### 3.4. Ingenuity Pathway Analysis (IPA) of SN Proteome

To obtain an in-depth understanding of the canonical pathways, disease associations, and upstream regulators driving neuropathic pain progression in the CCI model, Ingenuity Pathway Analysis (IPA) was performed using the DEP datasets from the SN. The top five significantly enriched canonical pathways based on z-scores included extracellular matrix (ECM) organization, SNARE signaling, collagen fibril assembly, neutrophil degranulation, and hepatic stellate cell activation (Figure S3). Immune-related signaling pathways such as MHC class II antigen presentation and interleukin signaling were also enriched (*p* < 0.05). Additionally, calcium signaling, neurotransmitter release, neural cell adhesion molecule (NCAM) signaling, and neurite outgrowth were prominently observed. Apart from these, pathways related to collagen biosynthesis, lipoprotein remodeling, and activation of liver X receptor/retinoid X receptor (LXR/RXR) were also significantly represented in the injured SN, indicating broad physiological remodeling.

IPA’s Disease and Function module revealed significant enrichment in pathways associated with neurological disorders, organismal injury, cardiovascular, and psychological functions. Sixteen functional molecular networks were identified, with the top network involving genes and proteins associated with metabolic disease, nervous system development, and neurological function (Table S1). Notably, ionotropic NMDA receptors (GRIN3A, GRIN1), PRKAR2A, PRKCE, and NDUFS4 were key molecules found within this network (Figure S4).

### 3.5. Gene Ontology and Pathway Analyses of the Exclusive Proteins Expressed in the SN of CCI Group

**Figure 3:**
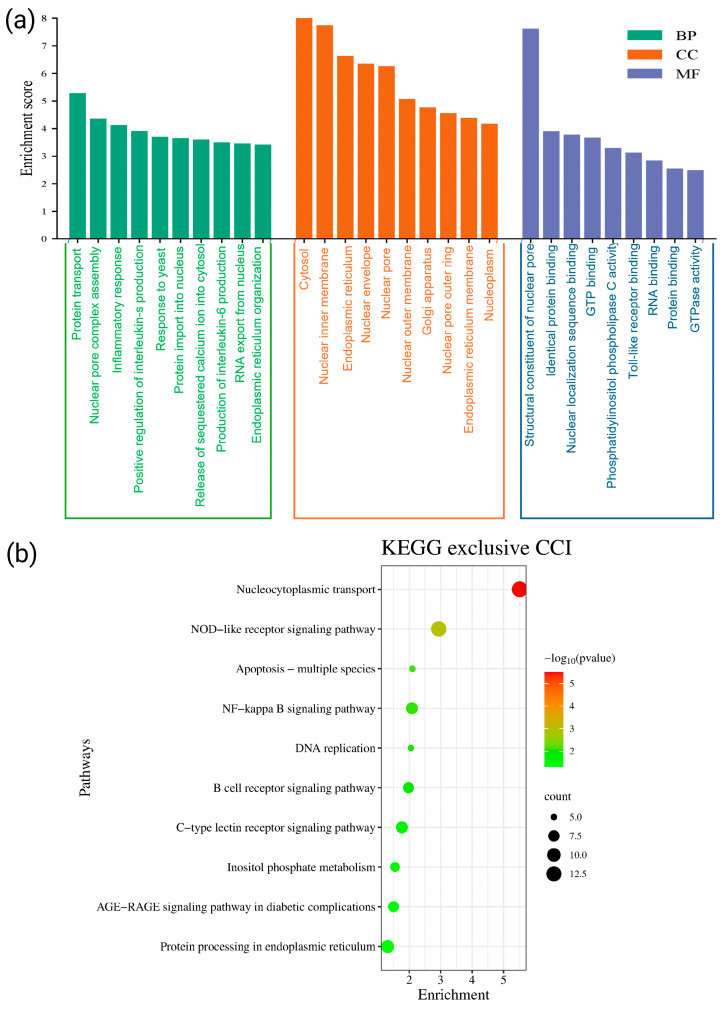
(**a**) The GO function analysis, i.e., biological process, cellular component, and molecular functions of the exclusive proteins expressed in the SN post-CCI. (**b**) The dot plot of the KEGG pathways of the exclusive proteins expressed in the SN of CCI rats. The horizontal axis represents the enrichment of the proteins, i.e., −log_10_ (*p* value) in the specific pathway, while the vertical axis represents pathways. The color scale indicates the different thresholds of the *p*-value and the size of dot represents the number of proteins involved in each term.

Proteomic profiling of the SN in CCI-induced NP rats revealed the expression of 4388 proteins, out of which 513 were found to be exclusively expressed in the CCI group. These unique proteins were subjected to GO and KEGG pathway enrichment analyses to uncover their biological relevance (Figure 3). GO analysis of biological processes (BP) showed significant enrichment in pathways related to inflammatory cytokine production, tumor necrosis factor (TNF-α) signaling, and positive regulation of NF-κB transcription factor activity (Figure 3a). These proteins were also associated with nucleocytoplasmic transport mechanisms, including nuclear pore complex (NPC) assembly. Additional BPs included cytosolic calcium release and phosphatidylinositol-mediated signaling. In the cellular component (CC) domain, the exclusive proteins were predominantly localized to cytosolic compartments, the endoplasmic reticulum (ER), nuclear envelope, Cajal bodies, and ER-to-Golgi vesicles (Figure 3a). For molecular functions (MF), enrichment was observed in nuclear localization sequence binding, RNA binding, Toll-like receptor binding, and phospholipase C activity, indicating their involvement in apoptosis and immune activation (*p* < 0.05 for all terms). Similarly, KEGG pathway analysis further showed that these proteins were involved in NOD-like receptor signaling, NF-κB and B cell receptor signaling, apoptosis, and inflammatory pathways such as AGE-RAGE and C-type lectin receptor signaling (Figure 3b). Collectively, these findings highlight that the exclusive protein signature in the SN following CCI injury encompasses critical regulators of inflammation, cell signaling, transcriptional modulation, and neuronal excitability, contributing to both the pathophysiology and adaptive responses to NP.

### 3.6. Quantification of Differentially Expressed Proteins (DEPs) and Identification of Up- and Down-Regulated Proteins in Spinal Cord

**Figure 4:**
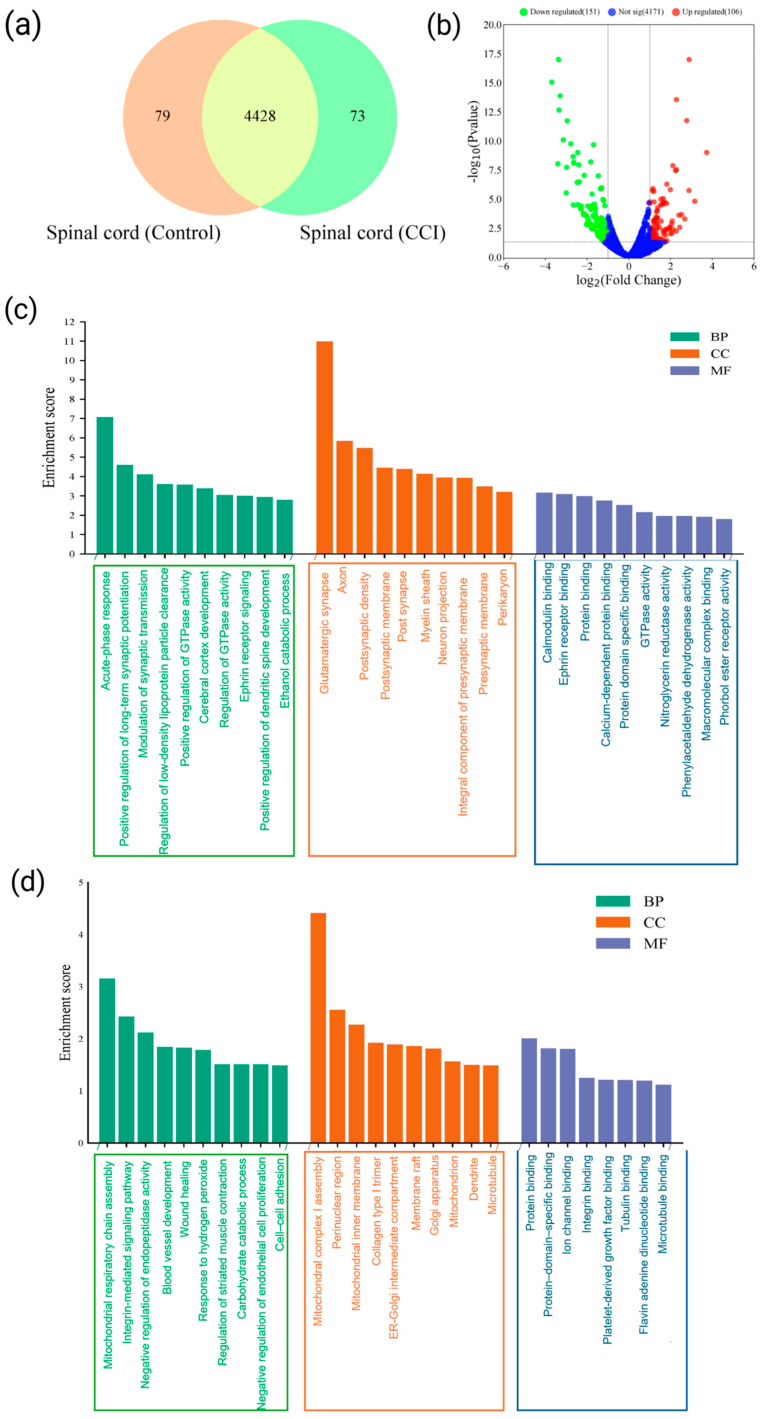

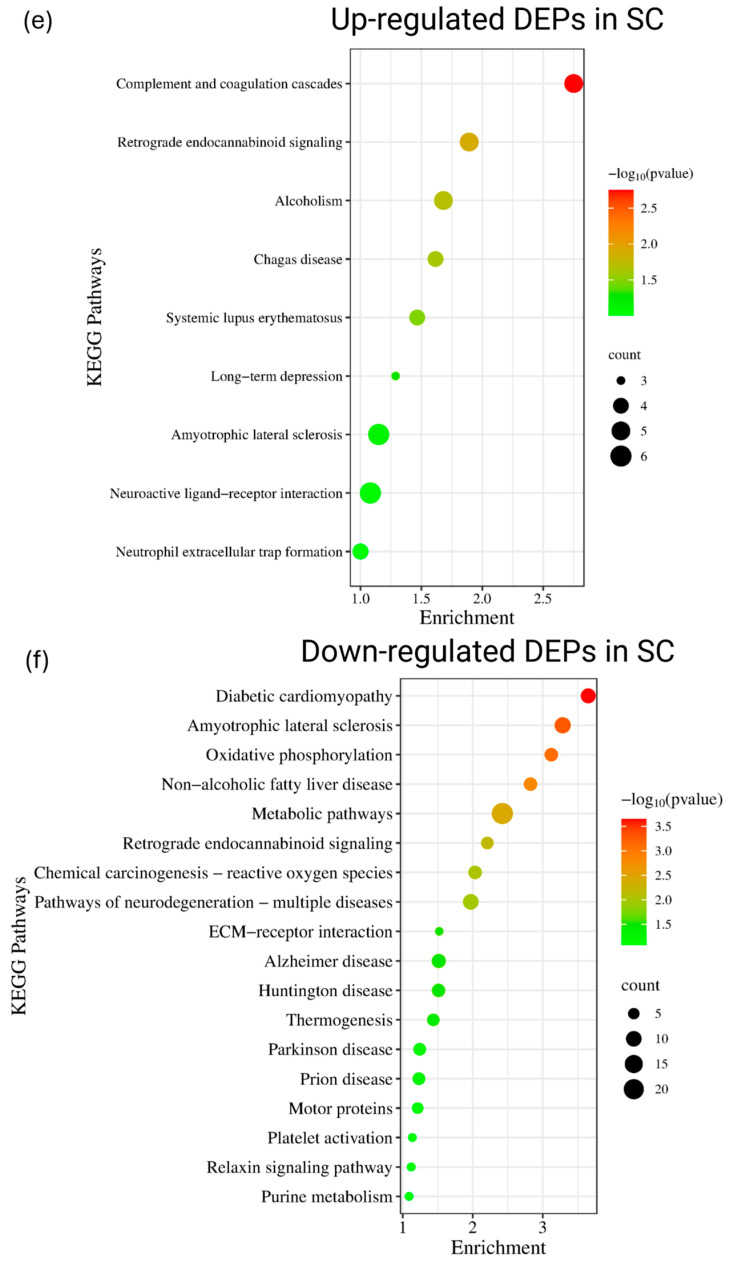
Identification and quantification of proteins expressed in the spinal cord. (**a**) Venn diagram representation of the overlapping proteins, i.e., DEPs between the CCI and control rats. (**b**) The volcano plot showing the significantly up- (red dot) and down-regulated (green dot) DEPs between both groups. The blue dot shows proteins with statistically insignificant differences between the groups. The *Y*-axis represents the negative logarithm of *p*-values, while the *X*-axis represents the average log2 (Fold changes) in protein expression. The bar plot representing the GO annotation of (**c**) up-regulated and (**d**) down-regulated proteins in SC post-injury. The dot plot representation of the KEGG pathways analysis enriched by (**e**) up-regulated and (**f**) down-regulated DEPs. The vertical axis represents the KEGG pathways and the horizontal axis represents enrichment, i.e., −log10 (*p*-value) of the associated pathway. The circles denote the count of proteins involved in a particular pathway.

Proteomic analysis of the SC in CCI-induced neuropathic pain (NP) rats revealed significant alterations in protein expression. A total of 4507 proteins were detected in control SC samples and 4501 in the CCI group. Among them, 4428 proteins (96.7%) were differentially expressed (Figure 4a). Within this set, 73 proteins were exclusively present in the CCI group, while 79 proteins were unique to the control group. Volcano plot analysis (Figure 4b) identified 106 significantly up-regulated and 151 significantly down-regulated proteins in the SC at 14 days post-CCI. These findings were confirmed by heatmap analysis (Figure S5), which revealed higher abundance of these proteins in the CCI group. Among the up-regulated proteins, notable candidates included gastrin-releasing peptide, T-kininogen 2, hippocalcin-like protein 4, S100A8, sequestosome-1, and syntaxin-binding protein 1 (Table 2). These proteins are implicated in pain perception, inflammation, calcium signaling, and synaptic transmission that promotes central sensitization directly or via the release of various inflammatory mediators. Conversely, the most significantly down-regulated proteins included sarcoplasmic/endoplasmic reticulum calcium ATPase-1, membrane-associated guanylate kinase (MAGUK), Aquaporin-1, and SH3 domain-binding protein 1. The above-mentioned proteins are associated with calcium homeostasis, spinal fluid regulation, cytoskeletal dynamics, and synaptic stability. Together, the proteomic landscape of the SC in CCI rats highlight a distinct proteomic alteration in the SC following CCI and suggest complex regulatory networks involving pro-inflammatory signaling, calcium dysregulation, synaptic remodeling, contributing to central sensitization and NP pathophysiology.

### 3.7. Functional Annotation of Up- and Down-Regulated Proteins Expressed in the Spinal Cord Post-CCI Injury

GO enrichment analysis was conducted on both up-regulated and down-regulated DEPs in the SC to assess CCI-induced functional remodeling (Figure 4c,d). Among the up-regulated DEPs, 94.2% were annotated under BP, 95.1% under CC, and 94.2% under MF. Similarly, the down-regulated DEPs also exhibited substantial GO coverage: 90.3% for BP, 88.8% for CC, and 76.9% for MF, indicating that peripheral nerve injury induces widespread proteomic shifts in the SC.

In the BP category, the up-regulated proteins were primarily enriched in processes related to positive regulation of long-term synaptic potentiation (LTP), synaptic transmission, dendritic spine morphogenesis, vesicle docking during exocytosis, ephrin receptor signaling, and oxidative stress response (Figure 4c). In the CC category, they were localized to glutamatergic synapses, dendritic spines, axons, myelin sheaths, and pre/post-synaptic membranes. In the MF ontology, they were involved in calcium-dependent binding (e.g., calmodulin and S100 family proteins), Eph-receptor binding, phospholipase inhibitor activity, and RAGE binding. In contrast, down-regulated DEPs were significantly enriched for BP terms such as mitochondrial respiratory chain complex I assembly, integrin-mediated signaling, hydrogen peroxide response, and carbohydrate catabolism (Figure 4d). Further, these proteins localized to components such as mitochondrial complex I, perinuclear cytoplasm, collagen type I trimer, Golgi apparatus, ER-Golgi intermediate compartment, and microtubules. In terms of MF, they were enriched for ion channel binding, integrin and tubulin binding, PDGF binding, FAD binding, and microtubule interactions.

Further, the KEGG pathway analysis was performed to investigate the biological pathways enriched among the up-regulated and down-regulated DEPs in the SC following CCI. The KEGG coverage was 55.3% for up-regulated DEPs and 54.5% for down-regulated DEPs. Among the up-regulated proteins, key enriched pathways included the complement and coagulation cascades, retrograde endocannabinoid signaling, and long-term depression, as well as pathways involved in Chagas disease, alcoholism, and systemic lupus erythematosus (*p* < 0.05; Figure 4e). Additionally, complement proteins such as complement component C9, α-2-macroglobulin, and complement factor B were also found to be significantly elevated, suggesting pronounced activation of innate immunity and a local surge in pro-inflammatory cytokines within the SC microenvironment following SN injury. In contrast, the down-regulated DEPs were primarily enriched in pathways related to oxidative phosphorylation, neurodegenerative diseases (e.g., Alzheimer’s, Parkinson’s, ALS, Huntington’s), prion disease, and diabetic cardiomyopathy (Figure 4f). These results indicate reduced activity in mitochondrial respiration and a broader suppression of neuroprotective and structural signaling pathways in the SC following peripheral nerve injury.

### 3.8. Ingenuity Pathway Analysis (IPA) of SC Proteome

IPA of the SC proteome following CCI revealed a highly interconnected molecular network governing the transition from peripheral nerve trauma to persistent NP, as illustrated in Figure S6. Canonical pathway mapping of both up-regulated and down-regulated DEPs identified five prominent pathways: GP1b-IX-V activation, NMDA receptor–mediated postsynaptic signaling, EGR2/SOX10-dependent Schwann cell myelination, platelet adhesion to exposed collagen, and extracellular matrix (ECM) reorganization (Table 3).

The top-ranked canonical pathway was GP1b-IX-V signaling, a platelet adhesion cascade activated by ECM disruption post-injury. This pathway facilitates phospholipase C activation, intracellular Ca^2+^ release, and increased chemokine production, resulting in the recruitment of immune cells to the SC. These infiltrating leukocytes interact with glial cells to enhance pro-inflammatory cytokine output, ultimately sensitizing nociceptive neurons and perpetuating pain signaling. Additional up-regulated pathways included ephrin B signaling and p75NTR receptor-mediated signaling, both implicated in neuroimmune modulation and neuronal plasticity. IPA revealed suppression of the GABAergic signaling pathway and activation of endocannabinoid synapse signaling in CCI rats. Notably, α-adrenergic signaling was also identified as a key mediator, contributing to ectopic neuronal firing and glial activation in the spinal neuraxis. Among these findings, the NMDA receptor-mediated postsynaptic signaling pathway was strongly enriched, with up-regulated subunits such as GRIN1 and GRIN3A.

Furthermore, to get more functional insight into the mapped proteins expressed in SC, we further analyzed the DEPs in the context of associated diseases and biofunctions. The analyses confirmed that the mapped DEPs in SC were associated with organismal injury along with neurological, cardiovascular, and gastrointestinal diseases. In terms of the physiological functions, these significantly expressed proteins are identified to be related to nervous system development along with other tissue repair (Table S2). One of the most notable networks proposed by IPA played a crucial role in studying neurological disease, organismal injury and abnormalities, and psychological disorders. Within this network, the molecules that play a crucial role in NP are BDNF, CAV1/2, CHRNA7, CNKSR2, CSF3, GABRG2, GNG7, GRIN subunits, KCNH5, MAP2, NECAB2, RNASE4, SLITRK1, SRC, SUMO2/3, and TNC (Figure S7).

### 3.9. Gene Ontology and Pathway Analyses of the Exclusive Proteins Expressed in the SC of CCI Group

Proteomic profiling of the SC fourteen days post-CCI identified 4501 proteins, of which 73 were exclusively expressed in the CCI group. The top ten exclusive proteins included S100 calcium binding protein A9, cytochrome c oxidase subunit 2, tumor necrosis factor receptor superfamily member 21, phospholipase A2, NADH dehydrogenase (Ubiquinone) 1 alpha subcomplex, Complement C8, protein S100-A3, dipeptidyl peptidase 9, apolipoprotein C-III, and exportin 7. Immunofluorescence confirmed S100 family protein expression in the SN, SC, and OFC of CCI rats, suggesting their up-regulation post-injury. Moreover, the presence of exportin-7 points to altered nuclear–cytoplasmic trafficking of microRNAs, potentially modulating injury-responsive gene expression.

**Figure 5:**
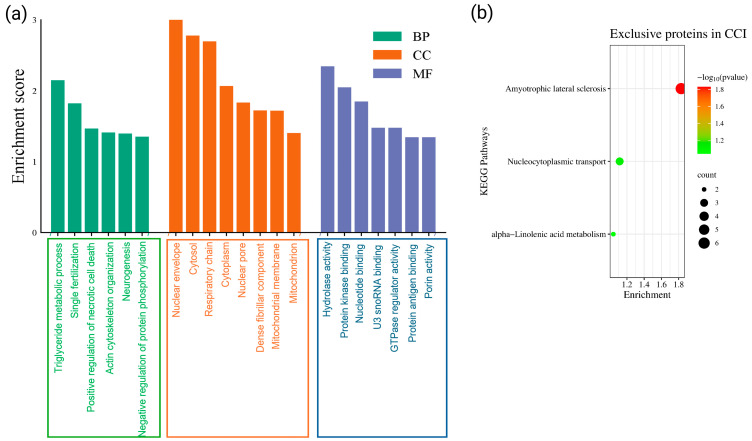
(**a**) The GO function analysis, i.e., biological process, cellular component, and molecular function of the exclusive proteins expressed in the spinal cord of CCI rats. (**b**) The dot plot of the KEGG pathways analysis enriched by up-regulated and down-regulated DEPs. The horizontal axis represents the enrichment of the proteins in the specific pathway, while the vertical axis represents pathways. The color scale indicates the different thresholds of the *p*-value and the size of dot represents the number of proteins on each term.

GO enrichment analyses of these 73 exclusive proteins revealed BP involved in triglyceride metabolism, necrotic cell death, and protein phosphorylation regulation. Other enriched BP terms included actin cytoskeleton organization, neurogenesis, and mitochondrial electron transport (Figure 5a). Comparably, CC analysis showed significant localization of these proteins to the cytosol, nuclear envelope, mitochondria, and kinetochore. Molecular function (MF) terms highlighted hydrolase activity, kinase binding, GTPase regulator activity, and porin activity, reflecting roles in membrane dynamics, signaling, and energy metabolism. These functions reflect the intersection of membrane dynamics, cytoskeletal remodeling, and lipid signaling in NP pathophysiology. Moreover, the potential and statistically significant KEGG pathways linked these exclusive proteins to inflammation-associated lipid signaling (Figure 5b).

### 3.10. Common Canonical Pathways Between SN and SC After the CCI of the Peripheral Nerve

To elucidate the shared molecular mechanisms sustaining NP along the proposed peripheral-central pain axis, proteomic analysis identified 300 canonical pathways common to both the SN and SC following CCI (Figure S8a,b). Among these, the top ten shared pathways included extracellular matrix (ECM) organization, clathrin-mediated endocytosis, glutamate receptor signaling, neutrophil extracellular trap formation, microautophagy signaling, ligand binding by scavenger receptors, serotonin receptor signaling, calcium signaling, p75 NTR receptor-mediated signaling, and SNARE signaling (Table 4). These shared pathways highlight the extensive molecular overlap between peripheral and central sites of injury.

### 3.11. Quantification of Differentially Expressed Proteins (DEPs) and Identification of Up- and Down-Regulated Proteins in Orbito Frontal Cortex (OFC)

**Figure 6:**
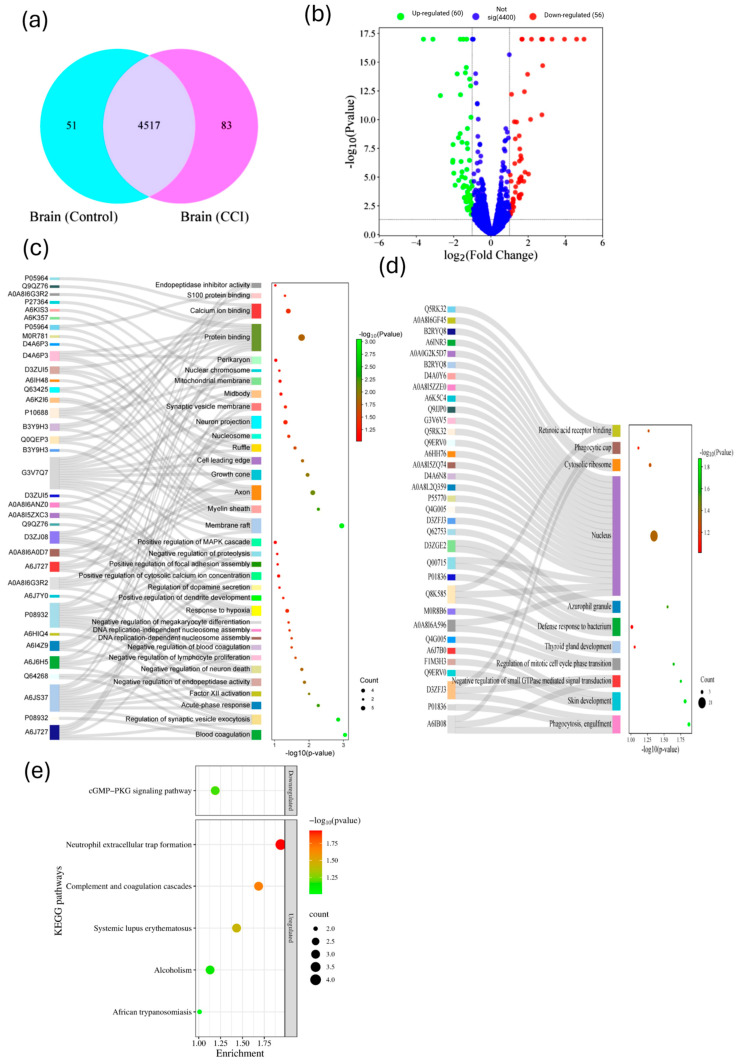
Identification and quantification of proteins expressed in the OFC. (**a**) Venn diagram representation of the DEPs between the CCI and control rats. (**b**) The volcano plot showing the up- (red dot) and down-regulated (green dot) proteins between CCI and control group. The blue dot shows proteins with statistically insignificant differences between the groups. The Y-axis represents the negative logarithm of *p*-values, while the X-axis represents the average log2 Fold changes in protein expression. Sankey plot representing the gene ontology (GO) annotation of up- (**c**) and down-regulated (**d**) proteins in the OFC of rats post-injury along with the proteins involved in the top 10 biological processes (BP), cellular component (CC), and molecular functions (MF). (**e**) KEGG pathways analysis enriched by up-regulated and down-regulated DEPs. The vertical axis represents the KEGG pathways. The horizontal axis is the enrichment, i.e., −log_10_ (*p*-value) of the associated pathway and vertical axis is the KEGG pathway. The circles denote the count of proteins involved in a particular pathway. The annotation of the accession ID of the up-regulated and down-regulated proteins in the OFC post-CCI has been mentioned in Supplementary Table S3.

Proteomic analysis of the OFC following CCI revealed substantial cortical proteomic remodeling, echoing the changes observed in the SC and SN. A total of 4568 and 4600 proteins were identified in the OFC of control and CCI rats, respectively, with 4517 proteins (97.1%) shared between the two groups (Figure 6a). Notably, 83 proteins (1.8%) were exclusive to the CCI group and 51 proteins (1.1%) to the control group, suggesting a profound proteomic shift induced by peripheral nerve injury. Volcano plot analysis revealed 56 significantly up-regulated and 60 down-regulated proteins in the CCI group (Figure 6b), and heatmap clustering showed consistent up-regulation across CCI samples (Figure S9).

Among the most upreulated proteins in OFC were RAD21, a core cohesin-complex subunit essential for chromatin loop formation and gene transcription; NADPH-dependent 3-ketosteroid reductase (AKR1C18), a key enzyme in neurosteroid biosynthesis; CD200, an immunomodulatory glycoprotein that regulates microglial reactivity; carbonic anhydrase 11 (CA11), which catalyzes the reversible hydration of CO_2_ and influences intracellular pH; T-kininogen 2 and kininogen, precursors of proinflammatory bradykinins; trinucleotide repeat containing 18 (TNRC18), involved in RNA processing; and non-receptor tyrosine phosphatase PTPN14, which modulates cytoskeletal and junctional dynamics (Table 5). In contrast, the down-regulated proteins included adhesion G protein-coupled receptor V1 (aGPCRs), histone H2B type 1, exostosin-like glycosyltransferase 2, NADH-ubiquinone oxidoreductase chain 2, and others.

### 3.12. Functional Annotation of Up- and Down-Regulated Proteins Expressed in the OFC Post-CCI Injury

To delineate the functional repercussions of peripheral nerve injury on supraspinal circuitry, we conducted comprehensive GO enrichment analyses on proteins whose expression was significantly altered in the OFC of rats fourteen days after CCI of the SN (Figure 6). In terms of the three GO categories that were covered by the up-regulated DEPs, the coverage was 92.5% for BP, CC, and MF. Likewise, the coverage of the GO categories for the down-regulated DEPs was 89.3% for BP, 92.9% for CC, and 82.1% for MF.

In terms of biological processes, the up-regulated proteins in OFC were predominantly clustered within processes governing synaptic function and cell survival. The most prominent BP terms included regulation of synaptic vesicle exocytosis, acute-phase response, negative regulation of endopeptidase activity, negative regulation of neuron death, and DNA replication-dependent nucleosome assembly. These processes implicate enhanced neurotransmitter release, inflammatory responses, protease regulation, neuroprotection, and chromatin reorganization. Additional enriched processes included blood coagulation, Factor XII activation, and immune-related BPs like negative regulation of megakaryocyte differentiation and lymphocyte proliferation, reflecting the body’s homeostatic attempts to modulate peripheral immune cell infiltration or glial activation post-CCI. Further, terms like “positive regulation of cytosolic calcium ion concentration”, “regulation of dopamine secretion”, and “positive regulation of MAPK cascade” revealed altered neurotransmission and intracellular signaling that underlie cortical excitability and synaptic plasticity, leading to persistent pain sensation. Similarly, CC enrichment among up-regulated proteins revealed localization to the myelin sheath, axon, growth cone, mitochondrial membrane, and other cytoskeletal structures indicative of synaptic remodeling, axonal sprouting, and metabolic adaptation post-CCI. Moreover, MF terms emphasized calcium ion binding and S100 protein binding, supporting roles in Ca^2+^ transients, kinase signaling, and presynaptic activity (Figure 6c). It is also important to note that S100 protein elevation was consistent across all three important tissues of the proposed pain neuraxis, i.e., SN, SC, and OFC, suggesting coordinated plasticity and inflammation across the pain neuraxis. In our datasets, we have also observed that there is up-regulation of proteins that regulate DA secretion in OFC.

In contrast, down-regulated DEPs in the OFC were enriched in BPs such as negative regulation of small GTPase-mediated signal transduction, regulation of mitotic cell cycle phase transition, and processes involving phagocytosis and defense response to bacteria (Figure 6d). Their associated cellular components included the nucleus, phagocytic cup, cytosolic ribosome, and azurophil granule. The most significant MF of the down-regulated DEPS in OFC was retinoic acid receptor (RAR) binding. Given RAR’s role in glial inhibition, its reduced expression post-CCI may exacerbate neuropathic phenotypes.

The coverage of up-regulated and down-regulated DEPs in the OFC from the datasets that were included in the KEGG database was 47.2% and 42.9%, respectively. Pathway analysis revealed that the up-regulated DEPs in the OFC were significantly enriched in inflammatory signaling pathways, including neutrophil extracellular trap formation and complement and coagulation cascades (Figure 6e). These pathways suggest persistent neuroinflammatory processes within the CNS triggered by chronic peripheral nerve injury. Such neuroinflammatory signaling may contribute to ongoing neuronal dysfunction and pain sensitization. Likewise, the down-regulated DEPs in OFC were primarily involved in the cGMP-PKG signaling pathway and down-regulation of these DEPs may impair anti-inflammatory responses and remove inhibitory control over pain-related circuits.

### 3.13. Ingenuity Pathway Analysis (IPA) of OFC Proteome

**Figure 7:**
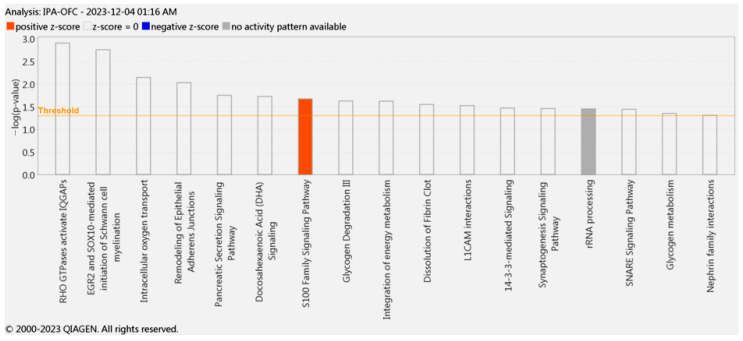
The top overlapping canonical pathways for the significantly up- and down-regulated DEPs expressed in the OFC post-CCI injury. The horizontal axis represents the canonical pathways and the vertical axis, i.e., log (*p*-value) line illustrates the “*p*-value of overlap” of the proteins in our dataset relative to IPA’s predefined canonicals.

IPA of the OFC proteome identified several canonical pathways and interaction networks critical to NP pathophysiology. The top five canonical pathways ranked by z-score were the following: RHO GTPases activate IQGAPs, ERG2 and SOX10-mediated initiation of Schwann cell myelination, intracellular oxygen transport, remodeling of epithelial adherens junctions, and pancreatic secretion signaling (Table 6). These pathways implicate actin cytoskeleton remodeling post-injury to the SN leading to IQGAP interaction with RHO GTPases, which can enhance TRPA1 membrane trafficking and promote cold and mechanical hypersensitivity. Additionally, DEPs in the OFC were involved in glycogen degradation, docosahexaenoic acid signaling, and L1CAM interactions. Interestingly, we also found that the proteins expressed in the OFC activated the S100 signaling pathway as similarly observed in other regions of the proposed algesic neuraxis (Figure 7).

More importantly, IPA identified seven functional networks that include DEPs from our datasets which can be utilized to forecast the molecular mechanisms in NP pathogenesis (Table S4). One of the most important networks that was postulated by IPA was found to be prominent in neurological disease, organismal injuries and abnormalities, and skeletal and muscle disorders. As a result, this network is most appropriate for NP pathophysiology, which is a neurological disorder induced by nervous system injury that results in motor impairments. This network is enriched in proteins involved in NP such as BDNF, CD200, CD388, CHRNA7, CHRNB2, EXTL2, and glutamate ionotropic receptor subunits (GRIN1, GRIN2A, GRIN2B, GRIN3A) (Figure S10) that are central to synaptic plasticity, immune modulation, and excitatory neurotransmission-all essential in NP development and maintenance.

### 3.14. Gene Ontology and Pathway Analyses of the Exclusive Proteins Expressed in the OFC of the CCI Group

**Figure 8:**
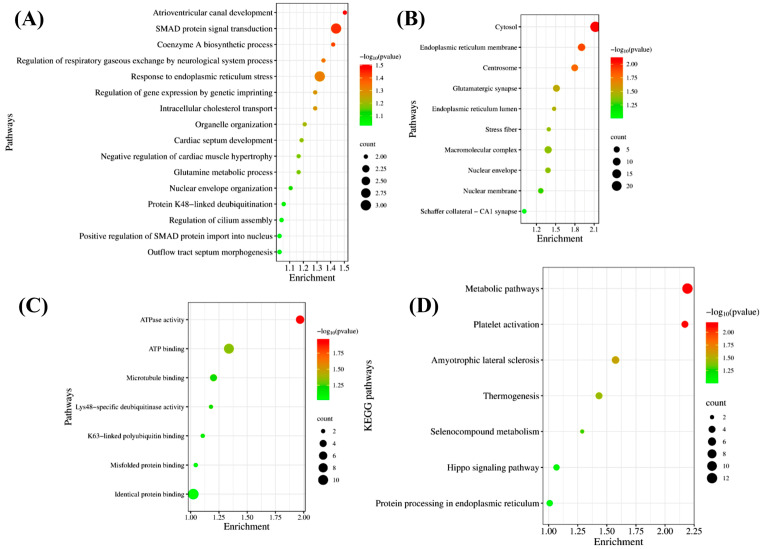
The dot plot of the GO function analysis, i.e., (**A**) biological process, (**B**) cellular component, and (**C**) molecular function of the exclusive proteins expressed in the OFC of CCI rats. The horizontal axis represents the enrichment of the proteins in the specific pathway, while the vertical axis represents pathways. The color scale indicates the different thresholds of the *p*-value and the size of dot represents the number of proteins on each term. The bubble map was constructed through the bioinformatics platform. (**D**) KEGG pathways analysis enriched by up-regulated and down-regulated DEPs. The vertical axis represents the KEGG pathways. The horizontal axis is the enrichment, i.e., −log_10_ (*p*-value) of the associated pathway and vertical axis is the KEGG pathway. The circles denote the count of proteins involved in a particular pathway.

Proteomic profiling of the OFC in rats fourteen days after CCI of the SN identified 4600 proteins, of which 83 were uniquely expressed in NP animals. Among the most abundant CCI-exclusive proteins were ATP synthase, kynurenine-oxoglutarate transaminase 3 (KAT3), periaxin, S100-A11, basal cell adhesion molecule, phosphopantothenoylcysteine decarboxylase, the acetyl-CoA transporter SLC33A1, arachidonate 15-lipoxygenase, and a complex in III-like protein. Their selective emergence lends credence to the hypothesis that they play a role in both the onset and progression of NP. Consequently, to elucidate the roles of these CCI-exclusive proteins, we performed GO and pathway enrichment analyses (Figure 8). BP-enriched among these proteins included glutamine metabolic process, response to endoplasmic reticulum stress, coenzyme A biosynthetic process, SMAD protein signal transduction, intracellular cholesterol transport, and protein K48-linked deubiquitination (Figure 8a). Likewise, the CC terms that are enriched for these proteins are mostly found in the endoplasmic reticulum membrane, glutamatergic synapse, nuclear envelope, centrosome, cytosol, and stress fiber (Figure 8b). Additionally, their MFs span ATPase activity, microtubule binding, Lys48-specific deubiquitinase activity, and K63-linked polyubiquitin binding (Figure 8c). Furthermore, the prospective and statistically significant KEGG pathways connected with these exclusive proteins have been linked to metabolic pathways, platelet activation, protein processing in the endoplasmic reticulum, and the Hippo signaling pathway (Figure 8d).

### 3.15. Comparative Canonical Pathway Analysis Between PNS and CNS in the Proposed Neuraxis

**Figure 9:**
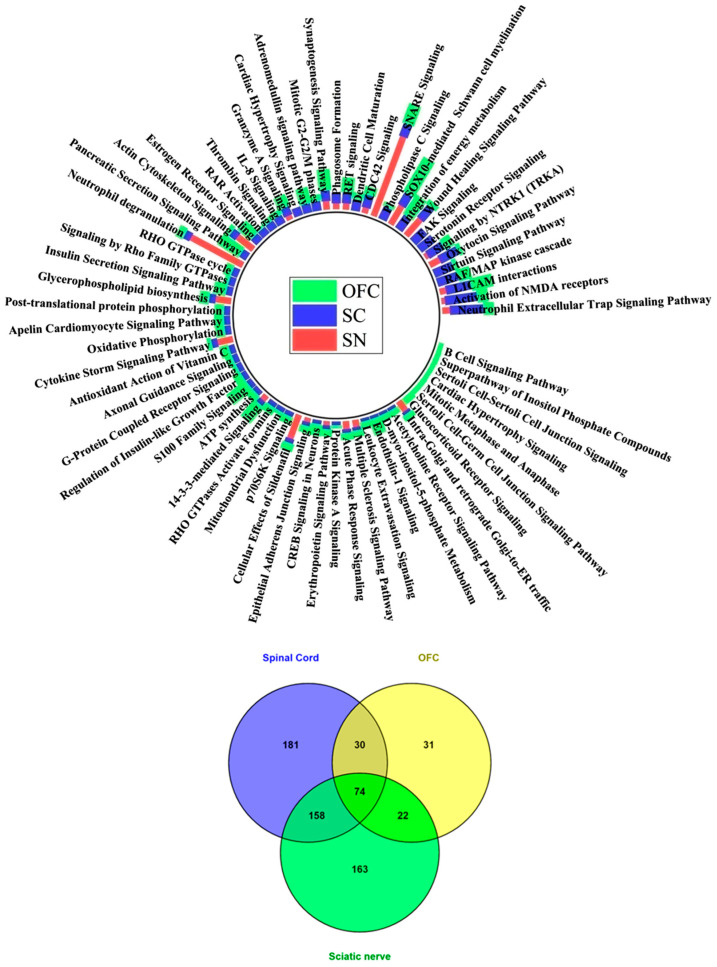
The comparative canonical pathways analysis between the sciatic nerve (SN), spinal cord (SC), and orbitofrontal cortex (OFC) identified by IPA. The height of the bar of the individual pathways is equivalent to the significance of the identified pathways (i.e., −Log (*p*-value; measured using the Fisher exact test)) in relation to the IPA reference canonical pathway.

Peripheral nerve injury studies have predominantly focused on the peripheral nerve and SC, while the role of supraspinal regions in pain perception has been comparatively understudied. In our study, we designed a framework to explore the proposed pain neuraxis, bridging the PNS and CNS, to investigate systemic alterations following CCI of the SN that contribute to the development and progression of NP. Comparative canonical pathway analysis across this axis revealed the enrichment of several common biochemical pathways, highlighting the molecular crosstalk between peripheral and central sites of pain processing (Figure 9). Inflammatory cascades were notably dominant throughout the neuraxis. Neutrophil extracellular trap formation, neutrophil degranulation, and S100-family signaling were among the most significantly enriched pathways across the SN, SC, and orbitofrontal cortex (OFC) (Table 7). Simultaneously, enrichment of SNARE-mediated exocytosis, synaptogenesis, and NMDA receptor signaling pathways across all three regions underscored their crucial role in excitatory neurotransmission and maladaptive plasticity. Mitochondrial dysfunction and activation of sirtuin pathways were also observed, suggesting systemic impairment in oxidative phosphorylation and cellular energy homeostasis. In contrast, up-regulation of endogenous analgesic mechanisms, including oxytocin and serotonin receptor signaling, was particularly evident in supraspinal regions, indicating compensatory responses to chronic pain.

### 3.16. CCI Induces Glial Cells Activation and S100 Protein Up-Regulation and Its Associated Neuroinflammation Across the Nociceptive Neuraxis

**Figure 10:**
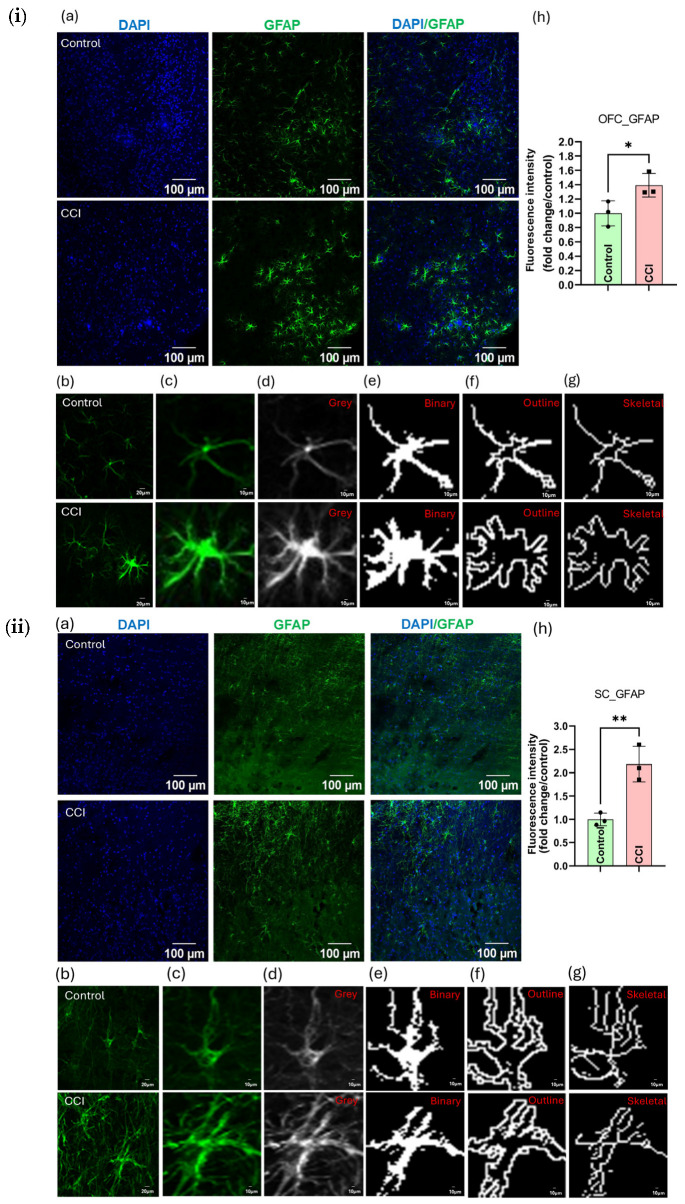
Astrogliosis in the ipsilateral (**i**) orbitofrontal cortex (OFC) and (**ii**) spinal cord (SC) following CCI. (**a**) Representative immunofluorescence micrographs from the ipsilateral OFC and SC showing DAPI (blue), GFAP (green), and merged images in control and CCI groups. Scale bar: 100 μm. (**b**,**c**) High-magnification images illustrating astrocyte morphology (20 and 10 μm scale bars respectively) followed by (**d**) grayscale conversion, (**e**) binary, (**f**) outline tracing, and (**g**) skeletonization. (**h**) Bar graphs (right side of panels) represent quantification of GFAP intensity. All values are in mean ± SD (*n* = 3 rats/group). To determine statistical significance, we employed two-tailed Student’s *t*-tests, with *p*-values of * *p* < 0.05, ** *p* < 0.01 indicating the levels of statistical significance.

Immunofluorescence analysis revealed significant activation of glial cells along the proposed nociceptive neuraxis post-CCI. Specifically, astrocyte activation was indicated by a marked increase in GFAP-positive cells in the OFC, SC, and SN of CCI rats compared to controls (*p* < 0.05) (Figure 10 and Figure S11). Higher magnification images showed that GFAP-positive astrocytes in CCI animals displayed hypertrophic morphology, with increased process thickness, branch points, elongation, and arborization complexity-confirmed via binary, outline, and skeleton maps in both OFC and SC (Figure 10i,ii, respectively).

**Figure 11:**
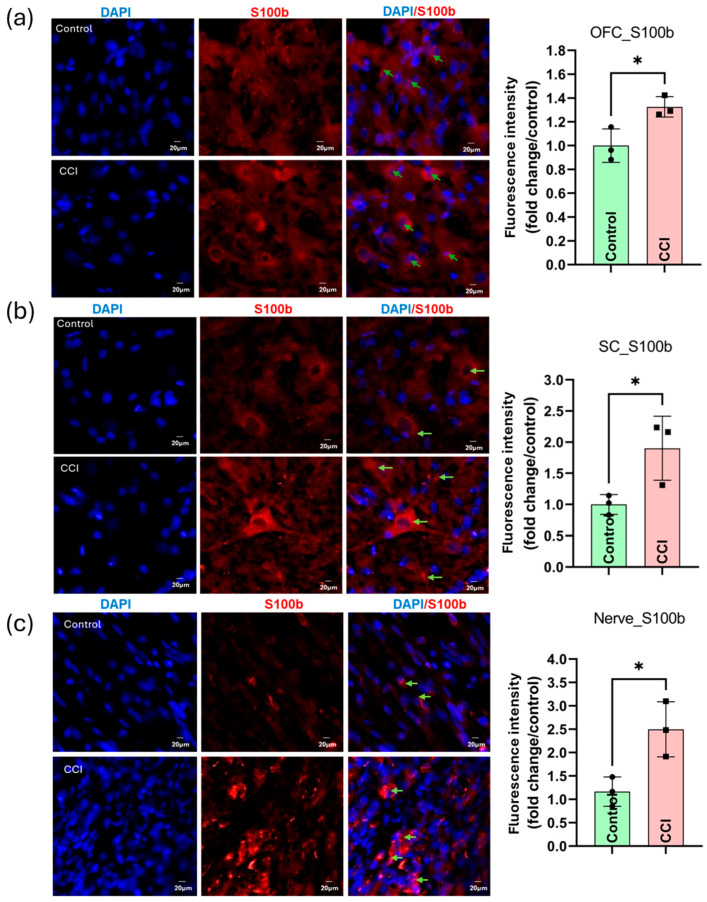
CCI-induced sciatic nerve injury leads to elevated S100b expression (green arrow) in OFC (**a**), ipsilateral dorsal horn of the SC (**b**), and ipsilateral sciatic nerve (**c**), indicating peripheral and central neuroinflammation. Scale bar was set at 20 μM with 40× magnification. All values are in mean ± SD (*n* = 3 rats/group). To determine statistical significance, we employed two-tailed Student’s *t*-tests, with *p*-values of * *p* < 0.05 indicating the levels of statistical significances.

Similarly, microglial activation was observed in both OFC and SC, as demonstrated by increased Iba-1 expression and morphological changes such as soma enlargement and process retraction (Figure S12). Structural analyses confirmed increased microglial complexity and reduced ramification in microglia of CCI rats (Figure S12c,f). Further, after the peripheral nerve injury, there was an elevated level of pro-inflammatory mediators (e.g., IL-6, TNF-α) in PNS and CNS as represented in Figure S13. Moreover, S100A8, a damage-associated molecular pattern (DAMP), was significantly up-regulated in the OFC, SC, and SN (*p* < 0.05; Figure S14). Additionally, S100B expression was strongly elevated across all three regions post-CCI, as shown by merged DAPI/S100B images and fluorescence quantification (Figure 11), supporting systemic neuroinflammatory responses (*p* < 0.05).

### 3.17. CCI of the Peripheral Nerve Enhances Dopaminergic Signaling in the CNS

**Figure 12:**
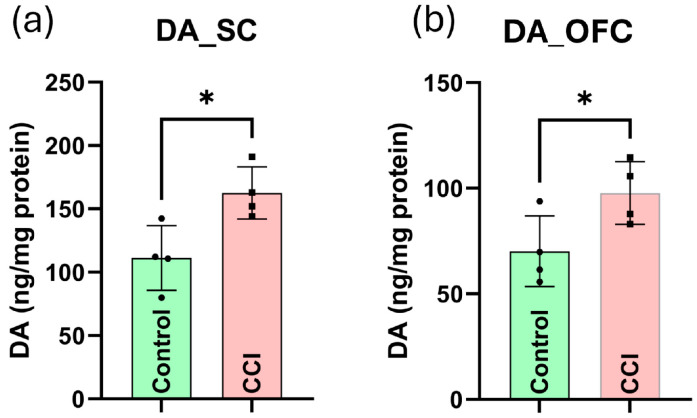
Effect of CCI of sciatic nerve on dopamine (DA) level in the (**a**) SC and (**b**) OFC. All values are in mean ± SD (*n* = four rats/group). To determine statistical significance, we employed two-tailed Student’s *t*-tests, with *p*-values of * *p* < 0.05 indicating the levels of statistical significance compared to control.

Following CCI, dopamine levels were significantly elevated in both the OFC and SC compared to control rats (Figure 12a,b), suggesting a neurochemical adaptation that may intersect with glial-mediated neuroinflammatory responses (*p* < 0.05). Additionally, the increased dopamine observed in the CCI model could represent either a maladaptive elevation contributing to neural sensitization or a homeostatic response attempting to engage endogenous analgesic circuits.

## 4. Discussion

In the present study, we characterized a nociceptive neuraxis linking the peripheral and central nervous systems in a CCI-induced NP in rats. Behavioral assessments demonstrated significant peripheral symptomatology, including the development of hyperalgesia, allodynia, motor incoordination, and muscle atrophy. Through quantitative proteomics, we identified widespread alterations in protein expression across the SN, SC, and OFC, revealing both region-specific and shared molecular signatures. Our key findings from the neuroproteomics study revealed up-regulation of inflammatory mediators, including S100 proteins, activation of astrocytes and microglia, altered neurotransmitter signaling, and enhanced synaptic plasticity. Further, the functional annotation and pathway analyses validated the involvement of neuroinflammatory and excitatory signaling cascades in NP pathogenesis. Additionally, enhanced dopaminergic signaling in the OFC emphasizes the engagement of affective and cognitive circuits in pain modulation. Overall, these results underscore the need for a system-level approach in comprehending NP and identifying novel therapeutic targets across the pain-processing axis.

The present study demonstrated the induction of NP phenotypes through CCI surgery, manifesting as thermal hyperalgesia, cold allodynia, mechanical hyperalgesia, and dynamic mechanical allodynia. These pain behaviors are consistent with earlier findings where CCI of the peripheral nerve leads to heightened sensory responses due to nociceptor sensitization and central disinhibition, the key drivers of enhanced sensory responses [50]. Reductions in withdrawal latency and increased response to thermal and cold stimuli suggest increased peripheral afferent excitability, especially in cold-sensing neurons [53], which gives an accurate representation of clinical symptoms such as cold allodynia in individuals with peripheral neuropathy. Additionally, the development of mechanical hyperalgesia and dynamic allodynia highlights the complex and overlapping interplay between spinal and supraspinal neuroplastic changes that lower the activation threshold of nociceptive circuits [54,55]. We also observed impaired motor function, evidenced by reduced rotarod performance, lower BBB scores, and a significantly diminished SFI, suggesting compromised motor neuron output and gait coordination [56]. These motor deficits mirror clinical neuropathies, which are often presented with impaired mobility [57]. Furthermore, significant muscle atrophy and sarcoplasmic disintegration, along with inflammatory infiltration and widened endomysium, were observed histologically in the gastrocnemius muscle, findings consistent with denervation and disuse-induced degradation [58]. This ultimately results in immobility or diminished usage of the affected limb, which decreases muscular protein synthesis and elevates protein degradation, leading to atrophy and a reduction in gastrocnemius muscle mass [58]. Consequently, these findings highlight the sensory and motor deficits induced by the CCI model, implicating the pathophysiology of NP and its associated musculoskeletal complications.

Quantitative proteomics revealed extensive molecular remodeling across the SN, SC, and OFC. In the SN, the up-regulation of inflammatory proteins, such as S100A8, and immune-modulatory proteins (e.g., translocon-associated protein subunit gamma, defensin NP-4), indicated persistent neuroinflammation. Notably, S100A8 emerged as the most highly expressed protein in the injured SN, consistent with earlier studies showing enrichment of the level of inflammatory markers post-peripheral nerve injury [59]. S100A8, a Ca^2+^ binding protein abundantly present in neutrophils and activated macrophages. It serves not only as a pro-inflammatory mediator but also as a ligand for the receptor for advanced glycation end products (RAGE), a signaling axis known to amplify neuroinflammation in both CNS and PNS [59,60]. The activated S100 proteins tend to modulate glial cell activation, triggering inflammatory responses through the release of pro-inflammatory cytokines and chemokines via a calcium-dependent pathway, hence contributing to the chronic nature of NP [61]. A recent study concurrently found that S100 protein was elevated in individuals suffering from trigeminal neuralgia, representing all of the symptoms observed in people suffering from NP [62]. Additionally, the translocon-associated protein subunit gamma, another significantly up-regulated protein in our dataset, resides in the membrane of the ER and is implicated in excessive loss of ER Ca^2+^. Loss of calcium homeostasis contributes to ER stress and subsequent excitotoxicity, a well-established mechanism in the perpetuation of NP [63]. Conversely, the concurrent down-regulation of MBP, kinases, and synaptic proteins indicates demyelination and impaired repair mechanisms that induce chronic NP symptoms [51,60,63,64]. Another significant observation was the down-regulation of the nuclear pore complex, a multiprotein channel that acts as a cargo for the transportation of macromolecules between the nucleus and cytoplasm [65]. Furthermore, they are also responsible for DNA repair following injury, as well as protein synthesis for restoration and maintenance, which was found to be reduced in our datasets of SN post-injury. Therefore, it can be asserted that peripheral nerve injury disrupts homeostatic regulatory systems, fostering an environment leading to the development of chronic pain.

Further, the GO and KEGG analyses identified biosynthetic activation (ribosomal biogenesis, cholesterol transport), metabolic stress, and inflammatory signaling (IL-17, NF-κB), while the IPA confirmed SNARE dysfunction and activation of interleukin, MHC-II, and NMDA receptor pathways [66,67]. Likewise, Hartlehnert et al. [68] demonstrated MHC class II up-regulation in Schwann cells post-trauma, contributing to axonal degeneration and heightened pain sensitivity. Additionally, consistent with previous clinical reports, disruption of ECM integrity was another prominent cellular pathway observed that drives maladaptive plasticity and promotes painful hypersensitive states [69]. Moreover, pathways involving the neural cell adhesion molecule (NCAM) and neurite outgrowth were enriched, which is reported to play an important role in synaptic plasticity and nociceptive hyperalgesia [70]. Apart from these, collagen biosynthesis, lipoprotein remodeling, and activation of liver X receptor/retinoid X receptor (LXR/RXR) receptors were enriched in the SN post-injury, which has been projected to modulate glial activation. Overall, the canonical pathways suggest that neuroinflammation, along with enhanced calcium signaling, is the major process that was prominent in SN post-CCI injury. Since the CCI animal model is a chronic injury model, the prediction by IPA confirms that post-injury there are statistically significant changes in the protein level that are involved in the disease pathogenesis and progression. Similarly, in the SC, the DEPs implicated central sensitization via ephrin-NMDA signaling, complement activation, and mitochondrial collapse [71]. Proteins such as T-kininogen 2, S100A8, and hippocalcin-like 4 emphasized calcium-dependent excitability and glial activation [72,73,74]. In support of our observation, Sun et al. [75] have exhibited that post-injury to the SC, there is enhanced expression of S100 proteins with the development of NP phenotypes like allodynia and hyperalgesia and pharmacological inhibition of S100 proteins reversed these nociceptive phenotypic behaviors [76]. Additionally, sequestosome-1, involved in autophagy, was also up-regulated in SC post-CCI of SN. Though autophagy generally dampens inflammation, excessive autophagosome formation in SC can activate microglia and aggravate pain phenotypes [55,77].

GO and KEGG analyses of the SC proteome revealed enhanced synaptic plasticity, cytoskeleton reorganization, and inflammation alongside suppression of oxidative phosphorylation and neuroprotection. Significantly, the concurrent down-regulation of mitochondrial complex I components and antioxidant pathways in both SC and SN indicated an axis-wide energy deficit and oxidative damage, contributing to neuronal hyperexcitability. In NP, it is known that mitochondrial dysfunction leads to excessive production of ROS and cytosolic Ca^2+^ imbalance [78,79]. Moreover, it also results in diminished ATP levels, which triggers glycolysis and lactate acidosis, exacerbating the persistence of ongoing pain [79]. Collectively, GO enrichment analysis delineates a dualistic response in the SC: up-regulated processes drive an “active” plastic and inflammatory state that facilitates central sensitization, while down-regulated pathways reflect a “passive” decline in metabolic and structural resilience that ordinarily constrains pathological excitability. Further, IPA highlighted GABAergic disinhibition, retrograde endocannabinoid signaling, ephrin B, NMDA, and p75NTR signaling, mirroring neuropathic and neurodegenerative pathways [71,80,81]. Endocannabinoids, notably 2-arachidonoylglycerol, act on presynaptic CB1 receptors to inhibit neurotransmitter release via modulation of Ca^2+^ influx at glutaminergic synapses [81,82]. Preclinical studies have demonstrated that exogenous endocannabinoid agonists attenuate hyperalgesia and allodynia by dampening excitatory transmission [81]. Our finding that proteins governing endocannabinoid biosynthesis, transport, and receptor interaction are up-regulated aligns with reports of elevated 2-arachidonoylglycerol levels in the SC and higher supraspinal regions post-CCI [83]. This trend in up-regulation of proteins likely represents an adaptive, homeostatic mechanism aimed at counteracting excessive excitatory signaling and mitigating pain sensitization. Together, these insights delineate a detailed framework of the molecular interplay, i.e., immune activation, excitatory plasticity, maladaptive myelination, inhibitory loss, and metabolic stress that overall underpins the persistence of NP following peripheral nerve injury.

In the OFC, exclusive DEPs such as RAD21, CA11, and AKR1C18 suggested transcriptional and neurosteroid alterations impairing GABAergic tone. Up-regulated CA and down-regulated K^+^-Cl^−^ cotransporters pointed to hyperexcitability [84,85] and disrupted pH regulation. Furthermore, accumulating evidence suggested that the pain processing strongly involves forebrain regions other than the somatosensory system, like the prefrontal cortex, amygdala, striatum, and OFC via the DA projections [25]. The DA neurons have been reported to have hemoglobin-like molecules (neuroglobin) that possess proton buffering properties due to the enzyme CA [86]. During NP, the up-regulation of CA causes excess production of HCO_3_^−^, which leads to intracellular acidification and modulation of TRP channels [87]. Aside from this, HCO_3_^−^-induced depolarization along with a simultaneous decrease in expression of K^+^-Cl^−^ co-transporter post-injury of SN promotes pain symptoms [84], contributing to loss of Cl^−^-dependent inhibitory neurotransmission and GABA_A_-mediated hyperexcitation [85]. In support of our observation, a report by Potenzieri et al. [87] has suggested that CA inhibitors reversed the cold allodynia in mice. Furthermore, GO and KEGG results also supported up-regulation of dopamine signaling, synaptic vesicle exocytosis, and inflammatory networks. The neuroinflammatory responses may further exacerbate neuronal damage and may contribute to the progression of NP. In support of our findings, clinical studies have demonstrated that neuroinflammation is the key pathological factor in the pathogenesis of NP [88]. Interestingly, in our study, we demonstrated dopamine elevation in both OFC and SC, suggesting a neurochemical adaptation intersecting with cytokine-mediated glial signaling, thereby engaging the emotional–cognitive dimension of NP [89]. This aligns with the reward theory of pain, which posits that mesocorticolimbic dopamine pathways, particularly projections to the OFC, integrate nociceptive inputs with reward and motivational processing, shaping both pain perception and its affective evaluation [24]. In the consonant of our findings, Scott et al. [90] reported elevated dopaminergic neurotransmission in the reward–motivation axis of the brain in response to noxious stimulation. Therefore, the OFC functions as a key integrative hub that assesses the aversive value of pain relative to potential rewards [91]. In contrast, studies have reported no changes in hedonic or motivational responses in animals with spinal nerve ligation-induced NP [92]. Moreover, the proposed regions in the “pain neuraxis” exhibited increased levels of neuroinflammatory markers (S100B and A8) together with astrocytic (GFAP) and microglial (Iba-1) proteins, which demonstrate the role of cortical regions in pain processing. Additionally, the elevated DA levels in SC and OFC demonstrate a coordinated shift of mesocorticolimbic pathways that could represent either adaptive or compensatory mechanisms for endogenous analgesia [25]. The dopaminergic modulation observed in the study may originate from glial-neuroimmune interactions, as glial-derived cytokines such as TNF-α and IL-6 modify dopamine transporter (DAT) function, leading to the development of NP phenotypes [89,93]. Likewise, the KEGG pathways highlighted the enrichment of DA signaling and DA metabolic enzymes, indicating dopaminergic adaptation across the entire neuraxis in response to chronic pain. Together, these findings suggest that chronic pain disrupts the reciprocal relationship between sensory input and reward processing, contributing to both heightened pain perception and its emotional burden.

## 5. Summary

The findings of this study advance our understanding of NP by conceptualizing a “nociceptive neuraxis” that bridges peripheral nerve injury with central neurobiological alterations. The proteomic landscape revealed across the SN, SC, and OFC underscores a coordinated inflammatory and neuromodulatory response, suggesting that NP is not solely a peripheral phenomenon but involves sustained central adaptations. The pronounced expression of S100 calcium-binding proteins and glial markers (GFAP and Iba-1) across these regions, along with elevated pro-inflammatory cytokines (TNF-α and IL-6), points to a neuroimmune axis that likely contributes to chronic pain. Simultaneously, protein enrichments linked to synaptic plasticity and dopamine signaling, particularly within the OFC, imply compensatory mechanisms that may influence affective dimensions of pain. These molecular alterations suggest that central structures involved in reward and emotion undergo adaptive changes in response to peripheral injury, offering insight into the emotional and cognitive burden of chronic pain. The identification of this integrated pain–processing axis offers a novel interpretive framework for understanding NP pathogenesis, emphasizing prominent molecular targets for interventions that concurrently modulate inflammatory, sensory, and emotional domains of chronic pain. These findings underscore the need for future studies to further elucidate the dynamic interplay between immune signaling and neurotransmission in the context of persistent nociception.

## Figures and Tables

**Table 1:** The top 10 up-regulated and down-regulated proteins expressed in the sciatic nerve post-CCI in rats.

Rank	Accession ID	Protein Description	Log_2_ (Fold Change)	*p*-Value
(A) Up-regulated proteins				
1	A6J6Q3	S100 calcium-binding protein A8 (Calgranulin A)	6.64	1 × 10^−17^
2	A6JIC0	Similar to RIKEN cDNA	6.13	1.73 × 10^−5^
3	A0A8I5Y616	Zinc finger protein 551	5.55	0.000169
4	F1M7T6	Translocon-associated protein subunit gamma	5.07	0.000184
5	A6IW99	Defensin NP-4	5.80	0.00022
6	A0A8I6GB30	NPC intracellular cholesterol transporter 1	5.81	0.000253
7	F1LNI5	Protein phosphatase 1G	4.90	0.000435
8	A6HZI4	Ribosomal protein S6 kinase, polypeptide 4	4.63	0.000668
9	D3ZJ08	Histone H3	4.53	0.000789
10	Q6P6T6	Cathepsin D	4.44	0.000987
(B) Down-regulated proteins				
1	A0A0G2JUP3	Non-specific serine/threonine protein kinase	−5.69	7.6 × 10^−9^
2	A0A0G2K313	LON peptidase N-terminal domain and ring finger 1	−5.73	8.74 × 10^−9^
3	A6KKG1	Albumin, isoform CRA_b	−4.33	2.58 × 10^−6^
4	A6K5J1	Myelin basic protein, isoform CRA_b	−4.13	3.14 × 10^−6^
5	A6KH67	Chymase 1, mast cell	−4.10	8.69 × 10^−6^
6	A6IB90	Nuclear pore membrane glycoprotein 210	−4.34	1.01 × 10^−5^
7	A0A0G2JZX4	Zinc finger protein 831	−4.21	2.5 × 10^−5^
8	A7M746	Keratin 83	−4.04	2.56 × 10^−5^
9	A6ICG2	Cysteine and glycine-rich protein 1, isoform CRA_b	−4.12	2.61 × 10^−5^
10	A6JKY8	RCG61079, isoform CRA_a	−4.38	2.62 × 10^−5^

**Table 2:** The top 10 up-regulated and down-regulated proteins expressed in the spinal cord post-CCI in rats.

Rank	Accession ID	Protein Description	*p*-Value
(A) Upregulated proteins			
1	P24393	Gastrin-releasing peptide	1 × 10^−17^
2	P08932	T-kininogen 2	1 × 10^−17^
3	A6JS37	RCG36716, isoform CRA_e	2.8 × 10^−14^
4	O08623	Sequestosome-1	1.79 × 10^−12^
5	A6JNL6	RCG31734 (Fragment)	1 × 10^−9^
6	P35332	Hippocalcin-like protein 4	1.36 × 10^−8^
7	Q04940	Neurogranin	2.95 × 10^−8^
8	A6J6Q3	S100 calcium binding protein A8 (Calgranulin A)	3.67 × 10^−8^
9	A6JU89	Syntaxin binding protein 1, isoform CRA_b	5.3 × 10^−7^
10	A6JFU2	Myelin protein zero, isoform CRA_a	1.28 × 10^−6^
(B) Down-regulated proteins			
1	Q64578	Sarcoplasmic/endoplasmic reticulum calcium ATPase 1	1 × 10^−17^
2	A0A8I6GMS2	Discs large MAGUK scaffold protein 1	8.88 × 10^−16^
3	A0A8I6A453	Myosin, light chain 1	1.33 × 10^−14^
4	P01836	Ig kappa chain C region, A allele	2.22 × 10^−13^
5	D3ZFJ3	SH3 domain-binding protein 1	1.9 × 10^−12^
6	A6K0X8	Aquaporin 1	8.2 × 10^−11^
7	A0A8I5ZMR5	TBC1 domain family member 9B	1.8 × 10^−10^
8	Q63011	Zero beta-globin (Fragment)	2.16 × 10^−10^
9	F1M529	Component of Sp100-rs	1 × 10^−9^
10	A0A8I6AQB7	Optic atrophy 3 (autosomal recessive, with chorea and spastic paraplegia)	2.21 × 10^−9^

**Table 3:** Top five canonical pathways with their *p*-values and the percentage of overlap of the expressed proteins from the datasets with the established proteins involved in the specific canonical pathways in the spinal cord post-CCI surgery.

Name of Canonical Pathways	*p*-Value	Overlap (%)	Accession ID
GP1b-IX-V activation signaling	9.80 × 10^−5^	33.3	COL1A1(collagen type 1; P02454) COL1A2 (collagen type 2; F1LS40) SRC (SRC proto-oncogene, non-receptor tyrosine kinase; Q9WUD9)
Activation of NMDA receptors and postsynaptic events	1.56 × 10^−4^	8.0	CAMK4 (calcium/calmodulin dependent protein kinase IV; A6J2R3) DLG1 (Discs large MAGUK scaffold protein 1; A0A8I6GMS2) GRIA2 (Glutamate ionotrophic receptor AMPA type subunit 2; P19491) RASGRF2 (Ras protein specific guanine nucleotide releasing factore2; Q99JE4) SRC (SRC proto-oncogene, non-receptor tyrosine kinase; Q9WUD9) TUBA8 (tubulin alpha 8; A0A8I5ZN97)
EGR2 and SOX10-mediated initiation of Schwann cell myelination	1.62 × 10^−4^	15.4	DRP2 (dystrophin related protein 2; A0A8L2R2Z3) MAG (myelin associated glycoprotein; A6JA35) MPZ (myelin protein zero; A6JFU2) PRX (periaxin; Q63425)
Platelet adhesion to exposed collagen	2.52 × 10^−4^	25.0	COL1A1 (collagen type 1; P02454) COL1A2 (collagen type 2; F1LS40) ITGA1 (Integrin subunit type 1; P18614)
Extracellular matrix organization	5.96 × 10^−4^	6.2	COL1A1 (collagen type 1; P02454) COL1A2 (collagen type 2; F1LS40) ITGA1 (Integrin subunit type 1; P18614) PTPRS (protein tyrosine phosphatase receptor type S; M0RA99) TNC (Tenascin C; B2LYI9)

**Table 4:** Top 10 common canonical pathways between sciatic nerve (SN) and spinal cord (SC) along with their *p*-values.

Canonical Pathways	SN	SC
Extracellular matrix organization	5.84	3.22
Clathrin-mediated Endocytosis	3.51	1.80
Glutamate receptor signaling	0.285	1.52
Neutrophil extracellular trap	0.514	1.24
Microautophagy signaling pathway	1.05	1.14
Binding ligands by scavenger receptors	2.23	2.31
Serotonin receptor signaling	0.02	1.04
Calcium signaling	2.66	1.19
p75 NTR receptor-mediated signaling	0.537	2.48
SNARE signaling	5.74	0.889

**Table 5:** The top 10 up-regulated and down-regulated proteins expressed in the orbitofrontal cortex (OFC) post-CCI in rats.

Rank	Accession ID	Protein Description	*p*-Value
(A) Up-regulated proteins			
1	A6JFZ1	Uncharacterized protein	1 × 10^−17^
2	A0A8I6ANZ0	RAD21 homolog (*S. pombe*)	1 × 10^−17^
3	P27364	NADPH-dependent 3-keto-steroid reductase Hsd3b5	1 × 10^−17^
4	A0A8I6A0D7	Cd200 molecule	1 × 10^−17^
5	Q811X3	Carbonic anhydrase 11	1 × 10^−17^
6	P08932	T-kininogen 2	1 × 10^−17^
7	Q6LE95	Kininogen	1 × 10^−17^
8	D3ZKV7	Trinucleotide repeat containing 18	1 × 10^−17^
9	A6JS37	RCG36716, isoform CRA_e	1 × 10^−17^
10	A0A8I6AFL3	protein-tyrosine-phosphatase	2 × 10^−15^
(B) Down-regulated proteins			
1	A0A096MK89	Adhesion G protein-coupled receptor V1	1 × 10^−17^
2	Q00715	Histone H2B type 1	1 × 10^−17^
3	F7F8T0	Exostosin-like glycosyltransferase 2	1 × 10^−17^
4	A0A8I6AZX8	Leucine rich repeat containing 75A	1 × 10^−17^
5	P01836	Ig kappa chain C region, A allele	1 × 10^−17^
6	Q06QH5	NADH-ubiquinone oxidoreductase chain 2	2.89 × 10^−15^
7	A0A8L2Q359	2-iminobutanoate/2-iminopropanoate deaminase	8.44 × 10^−15^
8	D3ZFJ3	SH3 domain-binding protein 1	1.04 × 10^−14^
9	A6JXS7	RCG32197, isoform CRA_a	2.95 × 10^−14^
10	A6JHP8	Similar to RIKEN cDNA 2810048G17 gene	1.16 × 10^−13^

**Table 6:** Top five canonical pathways with their *p*-values and the percentage of overlap of the expressed proteins in the OFC from the datasets with the established proteins involved in the specific canonical pathways in the OFC post-CCI surgery.

Name	*p*-Value	Overlap (%)	Accession ID
RHO GTPases activate IQGAPs	1.26 × 10^−3^	9.1	IQGAP1 (IQ motif containing GTPase activating protein1; G3V7Q7) TUBB1 (tubulin beta 1 class VI; M0R8B6)
ERG2 and SOX10-mediated initiation of Schwann cell myelination	1.76 × 10^−3^	7.7	ADGRV1 (Adhesion G protein coupled receptor V1; A0A096MK89) PRX (periaxin; Q63425)
Intracellular oxygen transport	7.18 × 10^−3^	33.3	MB (Myoglobin; Q9QZ76)
Remodeling of epithelial adherens junctions	9.39 × 10^−3^	3.3	IQGAP1 (IQ motif containing GTPase activating protein1; G3V7Q7) TUBB1 (tubulin beta 1 class VI; M0R8B6)
Pancreatic secretion signaling pathway	1.78 × 10^−2^	1.3	CA11 (Carbonic anhydrase 11; Q811X3) PLCD1 (phohpholipase C delta 1; P10688) STXBP2 (Syntaxin binding protein 2; Q62753)

**Table 7:** Top 10 canonical pathways between SN, SC, and OFC along with their *p*-values.

	Top 10 Canonical Pathways	SN	SC	OFC
1	Neutrophil Extracellular Trap Signaling Pathway	0.514	2.27	0.746
2	Activation of NMDA Receptors	0.236	2.21	0.782
3	Sirtuin Signaling Pathway	0.11	1.44	0.334
4	Oxytocin Signaling Pathway	0.747	1.38	0.329
5	Serotonin Receptor Signaling	0.03	1.27	0.12
6	Synaptogenesis Signaling Pathway	0.586	0.723	1.46
7	Neutrophil De-granulation	3.98	0.457	0.575
8	S100 Family Signaling	0.12	0.366	1.67
9	Mitochondrial Dysfunction	0.17	0.331	0.273
10	SNARE Signaling	5.74	0.889	1.44

## Data Availability

The raw data supporting the conclusions of this article will be made available by the authors on request.

## Supplementary Materials

The following supporting information can be downloaded at: https://www.mdpi.com/article/10.3390/cells15030290/s1, Figure S1: Identification and quantification of proteins expressed in the sciatic nerve. (a) Venn diagram representation of the overlapping proteins i.e., DEPs between the CCI and control rats. (b) The volcano plot showing the significantly up- (red dot) and down-regulated (green dot) DEPs between both the groups. The blue dot shows proteins with statistically insignificant differences between the groups. The Y-axis represents the negative logarithm of *p*-values, while the X-axis represents the average log_2_ (Fold changes) in protein expression; Figure S2: The abundances of the upregulated (C) and downregulated (D) proteins in the sciatic nerve of control and CCI rats along with their Uniprot Accession ID; Figure S3: The top overlapping canonical pathways for the significantly up and downregulated DEPs expressed in the SN post-CCI injury. The horizontal axis represents the canonical pathways and the vertical axis i.e., log (*p*-value) line illustrates the “*p*-value of overlap” of the proteins in our dataset relative to IPA’s predefined canonicals; Figure S4: Relationship between specific DEPs in SN and canonical pathway (neuropathic pain) based on the top network predicted by IPA. Red and green nodes indicate upregulated and downregulated proteins in the dataset, respectively. Brown and blue nodes indicate predicted activation ad inhibition of proteins, respectively by IPA.Akap9 (A-kinase anchoring protein 9), AMPH (amphiphysin), BDNF (brain-derived neurotrophic factor), CASP3 (Caspase 3), CDH10 (cadherin 10), CDK5 (cyclin dependent kinase 5), CDK5R (cyclin dependent kinase 5 regulatory subunit 1), CNKSR2 (connector enhancer of kinase suppressor of Ras 2), CRYAB (alpha-crystallin B), DCN (decorin), DDIT3 (DNA damage-inducible transcript 3), DLG2 (disks large homolog 2), DLG4 (disks large homolog 4), DNM1 (dynamin 1), GPNMB (glycoprotein NMB),GRIN1 (glutamate ionotrophic receptor NMDA type subunit 1), GRIN3A (glutamate ionotrophic receptor NMDA type subunit 3), GSK3B (glycogen synthase kinase-3 beta), HMOX1 (heme oxygenase 1), KCNA2 (Potassium voltage-gated channel subfamily A Member 2), KCNA4 (Potassium voltage-gated channel subfamily A Member 4, KIF5B (Kinesin-1 family member 5B), LGI1 (Leucine-rich glioma inactivated protein 1), MAPT (microtubule-associated protein tau), NDUFS4 (NADH dehydrogenase ubiquinone), PRKAR2A (cAMP-dependent protein kinase type-II-alpha regulatory subunit 2A), PRKCE (protein kinase C epsion), PTEN (phosphatase and tensin homolog), RB1 (retinoblastoma protein), SH3GL2 (Endophilin-A1), SNAP91 (synaptosome associated protein 91), SNCA (alpha-synuclein), STXBP1 (syntaxin binding protein 1), SYNJ1 (synaptojanin 1), and TSC (tuberous sclerosis protein). Solid lines indicate direct connections, while dotted lines indicate indirect connections (circular arrows mean influence itself). The pointed and blunt arrow heads represent activating and inhibitory relationships, respectively. Orange lines indicate activation, blue inhibition, yellow indicates that findings are inconsistent with the state of downstream molecule, and gray indicates no predicted effect; Figure S5: The abundances of the upregulated (C) and downregulated (D) proteins in the spinal cord of control and CCI rats along with their Uniprot Accession ID; Figure S6: The top overlapping canonical pathways for the significantly up and downregulated DEPs expressed in the spinal cord post-CCI injury. The horizontal axis represents the canonical pathways and the vertical axis i.e., log (*p*-value) line illustrates the “*p*-value of overlap” of the proteins in our dataset relative to IPA’s predefined canonicals; Figure S7: Relationship between specific DEPs in spinal cord and canonical pathway (neuropathic pain) based on the top network predicted by IPA. Brain-derived neurotrophic factor (BDNF), caveolin (CAV1 and CAV2), cholinergic receptor nicotinic alpha 7 subunit (CHRNA7), connector enhancer of kinase suppressor of ras 2 (CNKSR2), colony stimulating factor (CSF3), gamma-aminobutryic acid receptor (GABRG2), guanine nucleotide-binding protein (GNG7), glutamate ionotrophic receptor (GRIN1,GRIN2A,GRIN2B, and GRIN3A), voltage-gated potassium channel (KCNH5), microtubule associated protein (MAP2), N-terminal EF-hand calcium binding protein 2 (NECAB2), ribonuclease A family member 4 (RNASE4), SLITRK1, SRC proto-oncogene (SRC), small ubiquitin like modifier (SUMO2, SUMO3), and troponin c (TNC). Red and green nodes indicate upregulated and downregulated proteins in the dataset, respectively. Brown and blue nodes indicate predicted activation ad inhibition of proteins, respectively by IPA. Solid lines indicate direct connections; while dotted lines indicate indirect connections (circular arrows mean influence itself). The pointed and blunt arrow heads represent activating and inhibitory relationships, respectively. Orange lines indicate activation, blue inhibition, yellow indicates that findings are inconsistent with the state of downstream molecule, and gray indicates no predicted effect. Scale bar was set at 20 μM with 40× magnification. All values are in mean ± SD (*n* = 3 rats/group). To determine statistical significance, we employed two-tailed Student’s *t*-tests, with *p*-values of * *p* < 0.05 indicating the levels of statistical significance; Figure S8: (i) Venn diagram representation of the canonical pathways between the SN and SC post-CCI injury. (ii) The Comparative canonical pathways analysis between the SN and SC identified by IPA. The height of the bar of individual pathways is equivalent to the significance of the identified pathways (i.e., −Log (*p*-value); measured using the Fisher Exact test) in relation to the IPA reference canonical pathway; Figure S9: The abundances of the upregulated (C) and downregulated (D) proteins in the OFC of control and CCI rats along with their Uniprot Accession ID; Figure S10: Relationship between specific DEPs in the OFC and canonical pathway (neuropathic pain) based on the top network predicted by IPA. Brain-derived neurotrophic factor (BDNF), CD200 molecule, CD388 molecule, cholinergic receptor nicotinic alpha and beta subunit (CHRNA7 and CHRNB2), exostosin like glycosyltransferase 2 (EXTL2), and glutamate ionotrophic receptor (GRIN1, GRIN2A, GRIN2B, and GRIN3A). Red and green nodes indicate upregulated and downregulated proteins in the dataset, respectively. Brown and blue nodes indicate predicted activation ad inhibition of proteins, respectivelyby IPA.Solid lines indicate direct connections, while dotted lines indicate indirect connections (circular arrows mean influence itself). The pointed and blunt arrow heads represent activating and inhibitory relationships, respectively. Orange lines indicate activation, blue inhibition, yellow indicates that findings are inconsistent with the state of downstream molecule, and gray indicates no predicted effect; Figure S11: CCI-induced neuroinflammation in the sciatic nerve is associated with increased GFAP expression. Scale bar was set at 20 μM with 40× magnification. All values are in mean ± SD (*n* = 3 rats/group). To determine statistical significance, we employed two-tailed Student’s *t*-tests, with *p*-values of * *p* < 0.05 indicating the levels of statistical significance; Figure S12. Microglial activation in the ipsilateral (i) orbitofrontal cortex (OFC) and (ii) spinal cord (SC) following CCI. (a) Representative immunofluorescence micrographs from the ipsilateral OFC (i) and SC (ii) showing DAPI (blue), Iba-1 (green), and merged channels in control and CCI group. Scale bar: 100 μm. (b,c) High-magnification microglial images (20 and 10 μm scale bars respectively) followed by (d) grayscale conversion, (e) binary, (f) outline tracing, and (g) skeletonization used for morphological quantification of microglial ramification. (h) Bar graphs (right side of panels) represent quantification of Iba-1 intensity. All values are in mean ± SD (*n* = 3 rats/group). To determine statistical significance, we employed two-tailed Student’s *t*-tests, with *p*-values of * *p* < 0.05 indicating the levels of statistical significance; Figure S13: Effect of CCI on neuroinflammation (TNF-α and IL-6) in the sciatic nerve (a and d respectively), spinal cord (b and e respectively), and orbitofrontal cortex (c and f respectively). All values are in mean ± SD (*n* = 6 rats/group). To determine statistical significance, we employed two-tailed Student’s *t*-tests, with *p*-values of ** *p* < 0.05 indicating the levels of statistical significance; Figure S14: CCI-induced sciatic nerve injury leads to elevated S100A8 expression (red arrow) in OFC (a), ipsilateral dorsal form of the SC (b), and ipsilateral sciatic nerve (c), indicating peripheral and central neuroinflammation. Scale bar was set at 20 μM with 40× magnification. All values are in mean ± SD (*n* = 3 rats/group). To determine statistical significance, we employed two-tailed Student’s *t*-tests, with *p*-values of * *p* < 0.05 indicating the levels of statistical significance; Table S1: All the networks of the up and down-regulated proteins expressed in the sciatic nerve according to IPA; Table S2: All the networks of the up and down-regulated proteins in the spinal cord according to IPA; Table S3: The upregulated and downregulated proteins (with their Accession ID) expressed in the orbitofrontal cortex (OFC) post-CCI in rats; Table S4: All the networks of the up and down-regulated proteins in the OFC according to IPA.
