# Supplementary material for: Integrative Proteomics Reveal Neuroimmune and Dopaminergic Alterations Across the Nociceptive Neuraxis in Neuropathic Pain
Source: Cells. 2026 Feb 4;15(3):290. doi: 10.3390/cells15030290 (PMC12896955; doi:10.3390/cells15030290)
Supplement: Supplementary file 1 [file cells-15-00290-s001.zip › cells-4061789-supplementary.pdf]

Integrative Proteomics Reveal Neuroimmune and Dopaminergic Alterations Across the Nociceptive Neuraxis in Neuropathic Pain

Shreyasi Majumdar[1,2], Santosh Kumar Prajapati[1,3], Aishwarya Dande[4], Vinod Kumar Yata[5], Khushboo Choudhary[4], Ramalingam Peraman[4], Nitesh Kumar[4], Sairam Krishnamurthy[1]*

[1]Neurotherapeutics Laboratory, Department of Pharmaceutical Engineering and Technology, Indian Institute of Technology (Banaras Hindu University), Varanasi-221005, India

[2]Department of Pharmaceutical Technology, School of Health and Medical Sciences, Adamas University, Kolkata-700126, India

[3]Department of Neurosurgery and Brain Repair, University of South Florida, Tampa, FL 33613, USA.

[4]National Institute of Pharmaceutical Education and Research, Hajipur-844102, India

[5]Department of Pharmacology, School of Allied Healthcare Sciences, Malla Reddy University, Hyderabad-500100, India

Number of Supplementary Figures: 14

Number of Supplementary tables: 04

*<u>**Corresponding author:**</u>

Dr. Sairam Krishnamurthy

Professor in Pharmacology

Neurotherapeutics Lab, Department of Pharmaceutical Engineering and Technology

Indian Institute of Technology (Banaras Hindu University)

Varanasi-221005, India

E-mail: ksairam.phe@iitbhu.ac.in

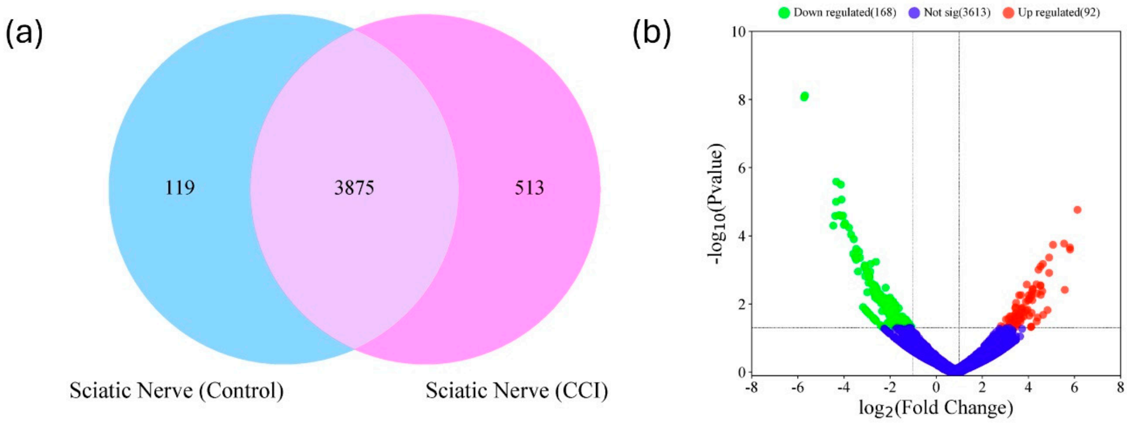

Figure S1: Identification and quantification of proteins expressed in the sciatic nerve. *(a)* Venn diagram representation of the overlapping proteins i.e., DEPs between the CCI and control rats. *(b)* The volcano plot showing the significantly up- (red dot) and down-regulated (green dot) DEPs between both the groups. The blue dot shows proteins with statistically insignificant differences between the groups. The Y-axis represents the negative logarithm of p-values, while the X-axis represents the average $\log_2$ (Fold changes) in protein expression.

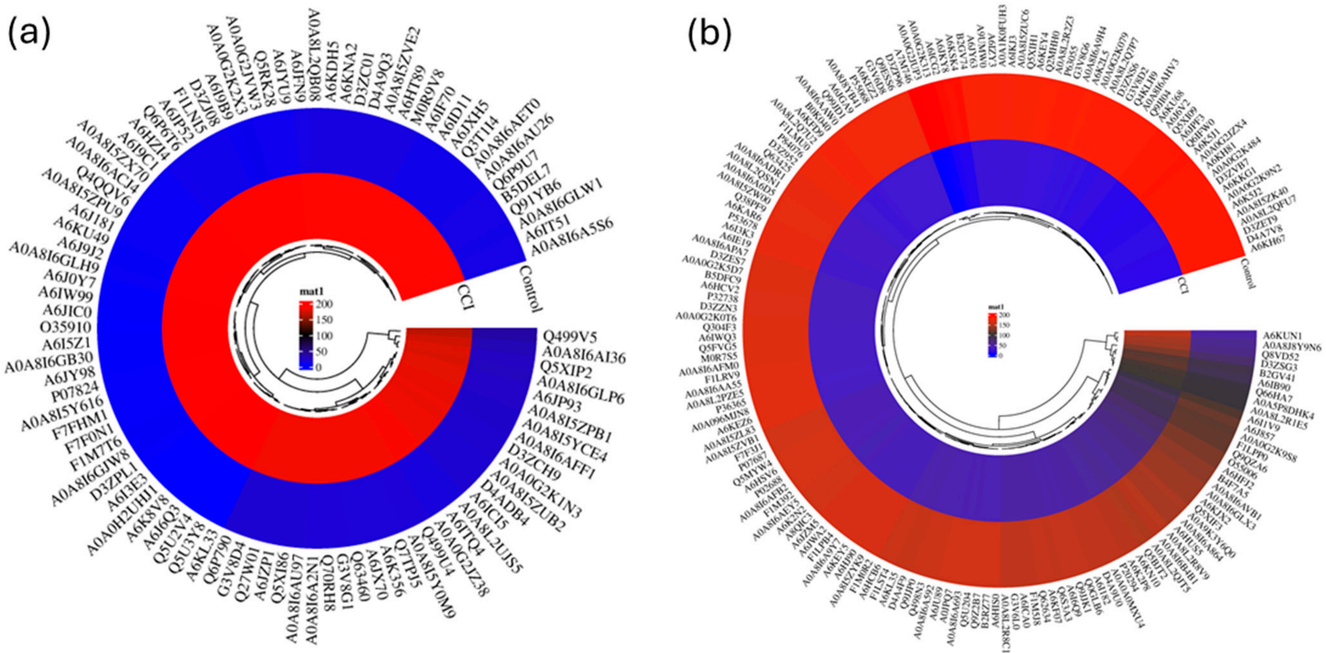

Figure S2: The abundances of the upregulated (C) and downregulated (D) proteins in the sciatic nerve of control and CCI rats along with their Uniprot Accession ID.

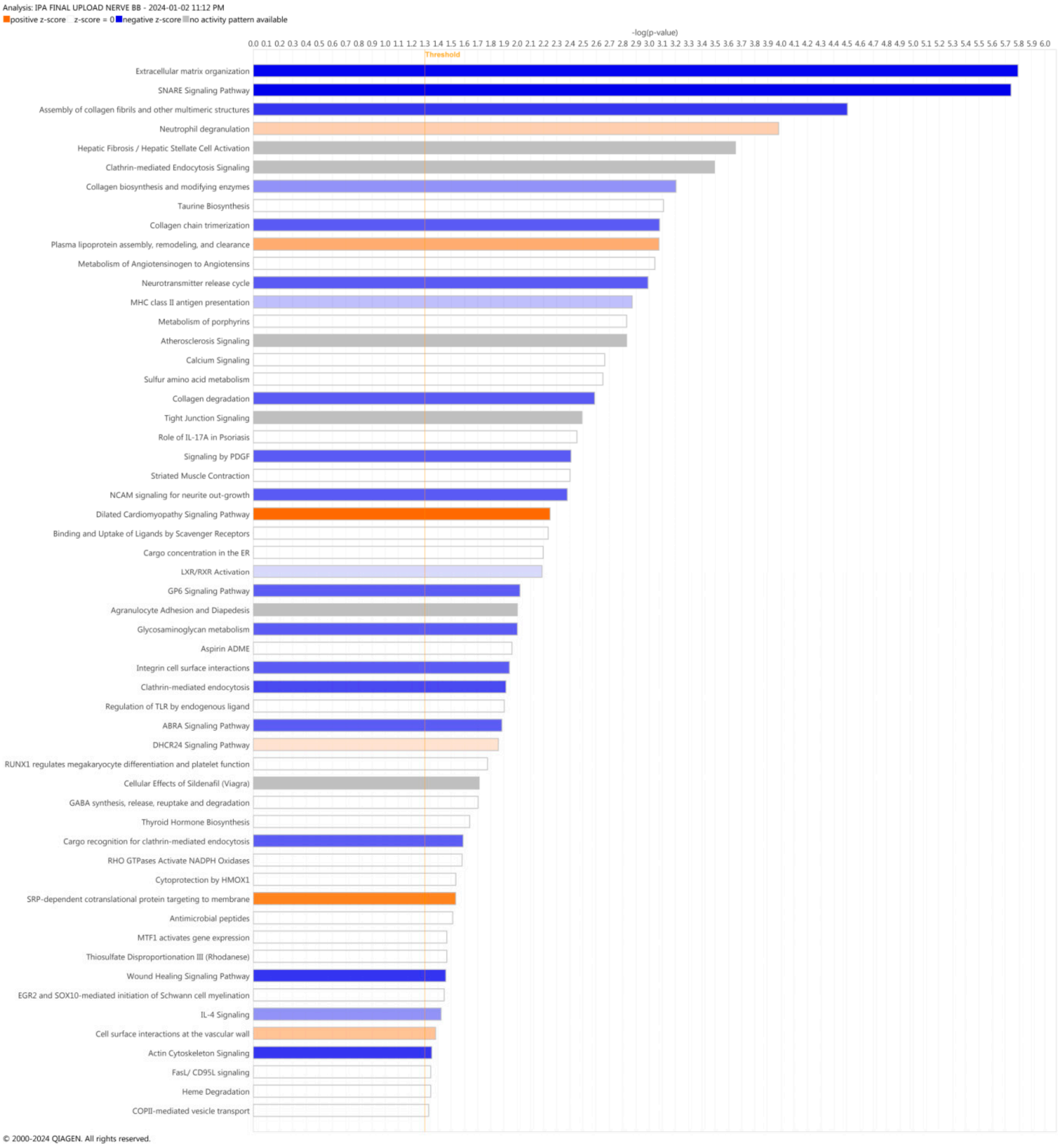

Figure S3: The top overlapping canonical pathways for the significantly up and downregulated DEPs expressed in the SN post-CCI injury. The horizontal axis represents the canonical pathways and the vertical axis i.e., log(p-value) line illustrates the "p-value of overlap" of the proteins in our dataset relative to IPA's predefined canonicals.

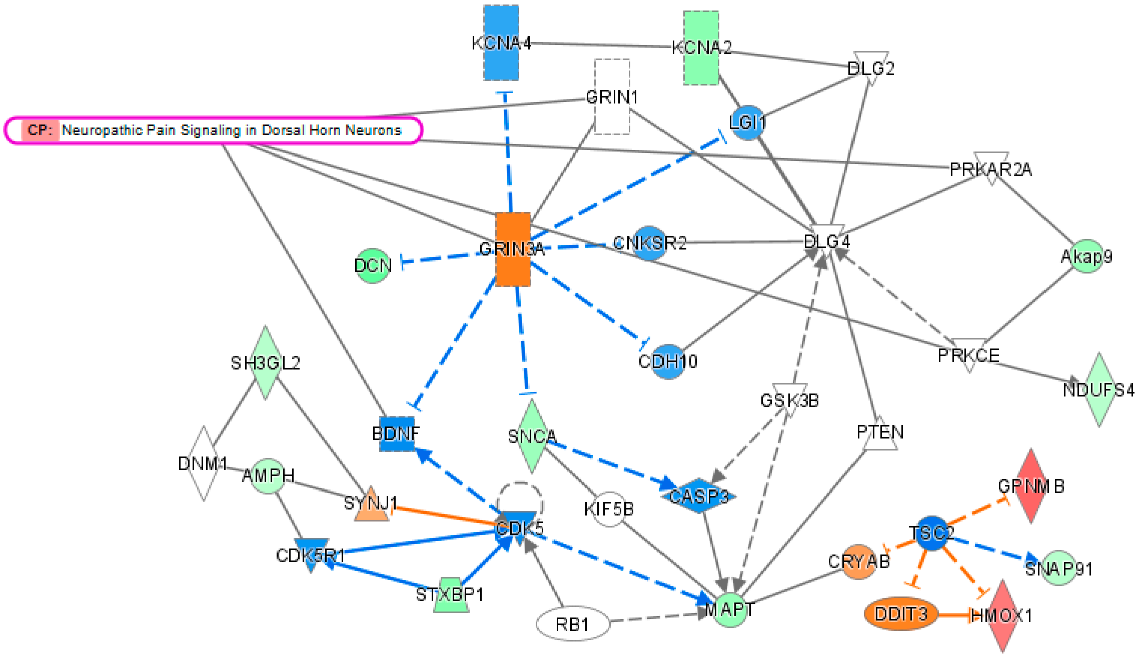

Figure S4: Relationship between specific DEPs in SN and canonical pathway (neuropathic pain) based on the top network predicted by IPA. Red and green nodes indicate upregulated and downregulated proteins in the dataset, respectively. Brown and blue nodes indicate predicted activation ad inhibition of proteins, respectively by IPA.Akap9 (A-kinase anchoring protein 9), AMPH (amphiphysin), BDNF (brain-derived neurotrophic factor), CASP3 (Caspase 3), CDH10 (cadherin 10), CDK5 (cyclin dependent kinase 5), CDK5R (cyclin dependent kinase 5 regulatory subunit 1), CNKSR2 (connector enhancer of kinase suppressor of Ras 2), CRYAB (alpha-crystallin B), DCN (decorin), DDIT3 (DNA damage-inducible transcript 3), DLG2 (disks large homolog 2), DLG4 (disks large homolog 4), DNM1 (dynamin 1), GPNMB (glycoprotein NMB),GRIN1 (glutamate ionotrophic receptor NMDA type subunit 1), GRIN3A (glutamate ionotrophic receptor NMDA type subunit 3), GSK3B (glycogen synthase kinase-3 beta), HMOX1 (heme oxygenase 1), KCNA2 (Potassium voltage-gated channel subfamily A Member 2), KCNA4 (Potassium voltage-gated channel subfamily A Member 4, KIF5B (Kinesin-1 family member 5B), LGI1 (Leucine-rich glioma inactivated protein 1), MAPT (microtubule-associated protein tau), NDUFS4 (NADH dehydrogenase ubiquinone), PRKAR2A (cAMP-dependent protein kinase type-II-alpha regulatory subunit 2A), PRKCE (protein kinase C epsion), PTEN (phosphatase and tensin homolog), RB1 (retinoblastoma protein), SH3GL2 (Endophilin-A1), SNAP91 (synaptosome associated protein 91), SNCA (alpha-synuclein), STXBP1 (syntaxin binding protein 1), SYNJ1 (synaptojanin 1), and TSC (tuberous sclerosis protein). Solid lines indicate direct connections, while dotted lines indicate indirect connections (circular arrows mean influence itself). The pointed and blunt arrow heads represent activating and inhibitory relationships, respectively. Orange lines indicate activation, blue inhibition, yellow indicates that findings are inconsistent with the state of downstream molecule, and gray indicates no predicted effect.

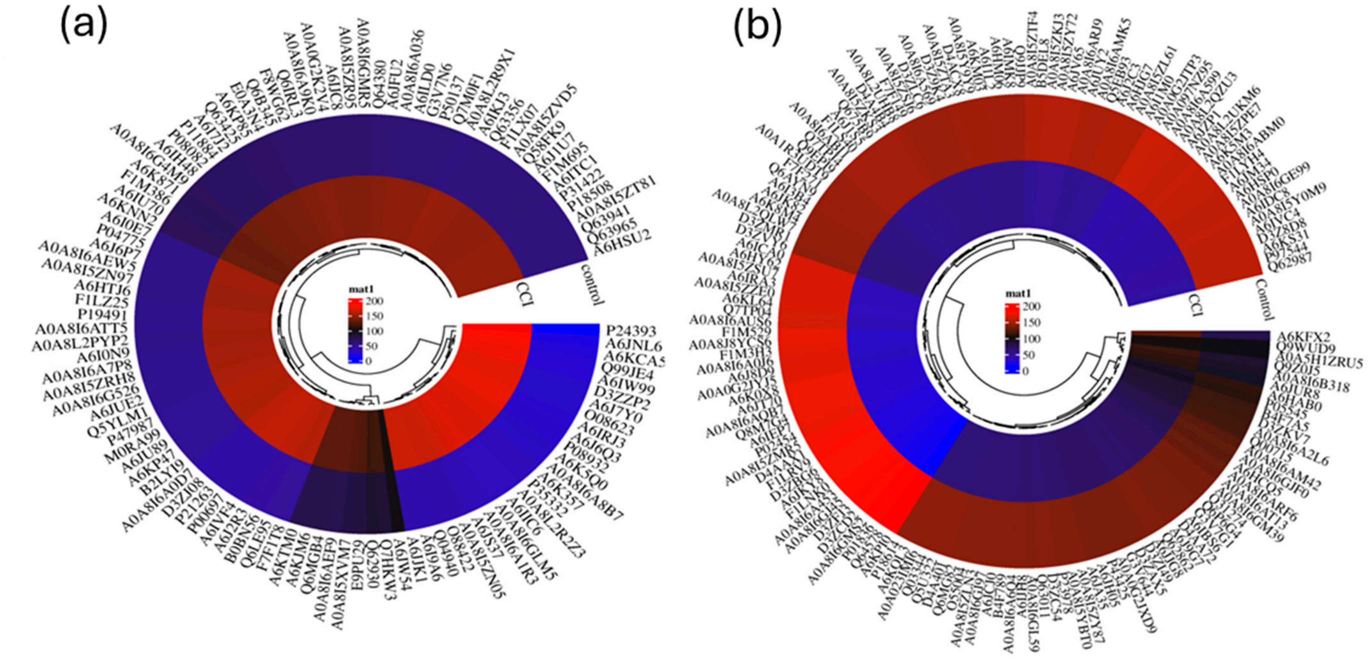

Figure S5: The abundances of the upregulated (C) and downregulated (D) proteins in the spinal cord of control and CCI rats along with their Uniprot Accession ID.

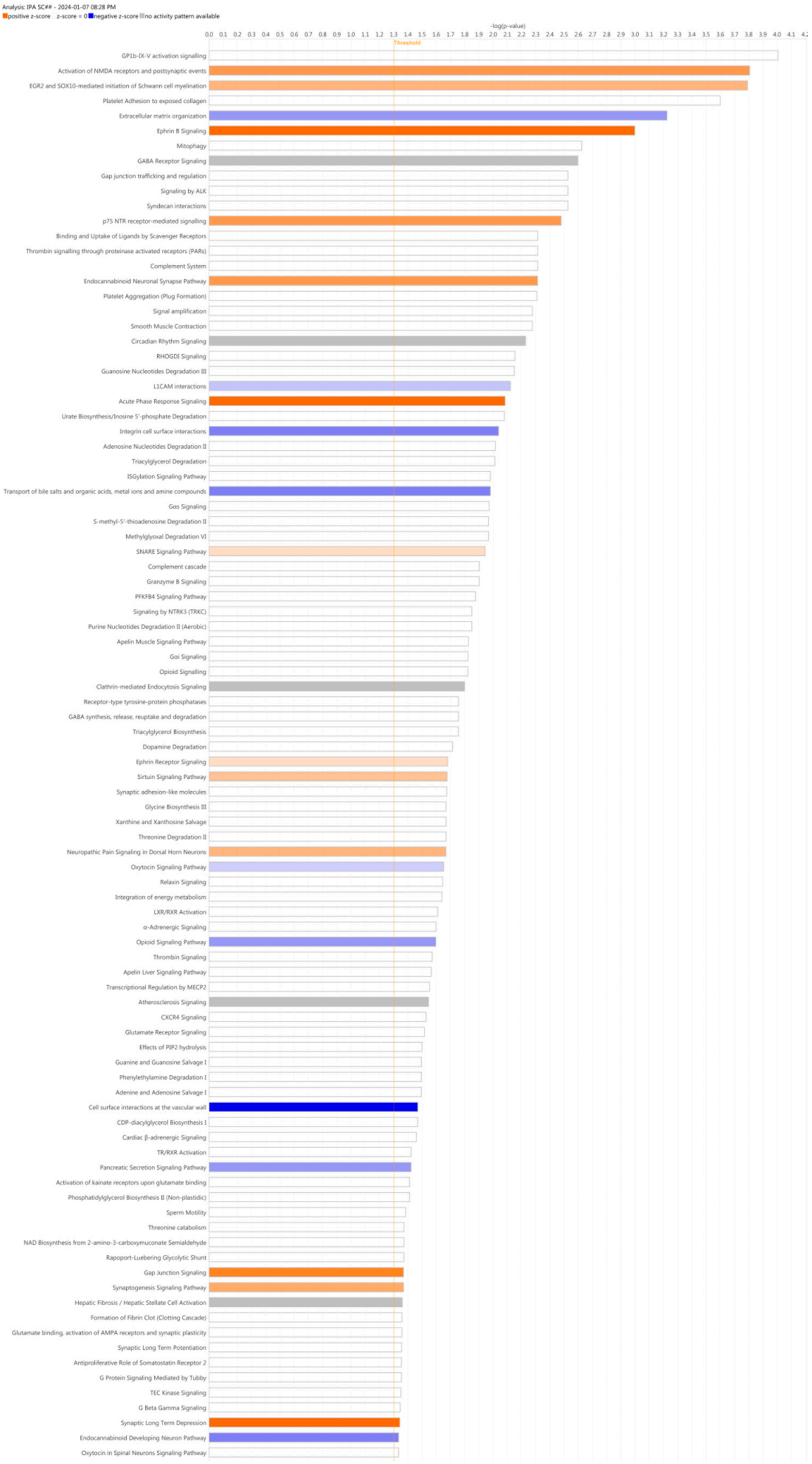

Figure S6: The top overlapping canonical pathways for the significantly up and downregulated DEPs expressed in the spinal cord post-CCI injury. The horizontal axis represents the canonical pathways and the vertical axis i.e.,

log(p-value) line illustrates the "p-value of overlap" of the proteins in our dataset relative to IPA's predefined canonicals.

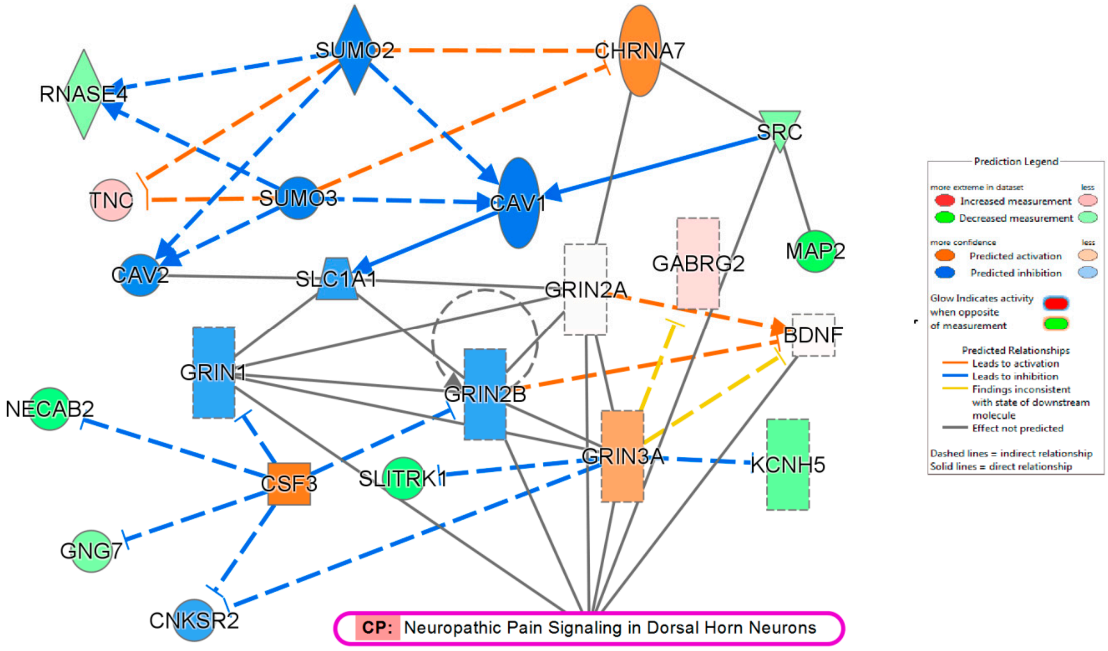

Figure S7: Relationship between specific DEPs in spinal cord and canonical pathway (neuropathic pain) based on the top network predicted by IPA. Brain-derived neurotrophic factor (BDNF), caveolin (CAV1 and CAV2), cholinergic receptor nicotinic alpha 7 subunit (CHRNA7), connector enhancer of kinase suppressor of ras 2 (CNKSR2), colony stimulating factor (CSF3), gamma-aminobutryic acid receptor (GABRG2), guanine nucleotide-binding protein (GNG7), glutamate ionotrophic receptor (GRIN1,GRIN2A,GRIN2B, and GRIN3A), voltage-gated potassium channel (KCNH5), microtubule associated protein (MAP2), N-terminal EF-hand calcium binding protein 2 (NECAB2), ribonuclease A family member 4 (RNASE4), SLITRK1, SRC proto-oncogene (SRC), small ubiquitin like modifier (SUMO2, SUMO3), and troponin c (TNC) Red and green nodes indicate upregulated and downregulated proteins in the dataset, respectively. Brown and blue nodes indicate predicted activation ad inhibition of proteins, respectivelyby IPA. Solid lines indicate direct connections, while dotted lines indicate indirect connections (circular arrows mean influence itself). The pointed and blunt arrow heads represent activating and inhibitory relationships, respectively. Orange lines indicate activation, blue inhibition, yellow indicates that findings are inconsistent with the state of downstream molecule, and gray indicates no predicted effect.

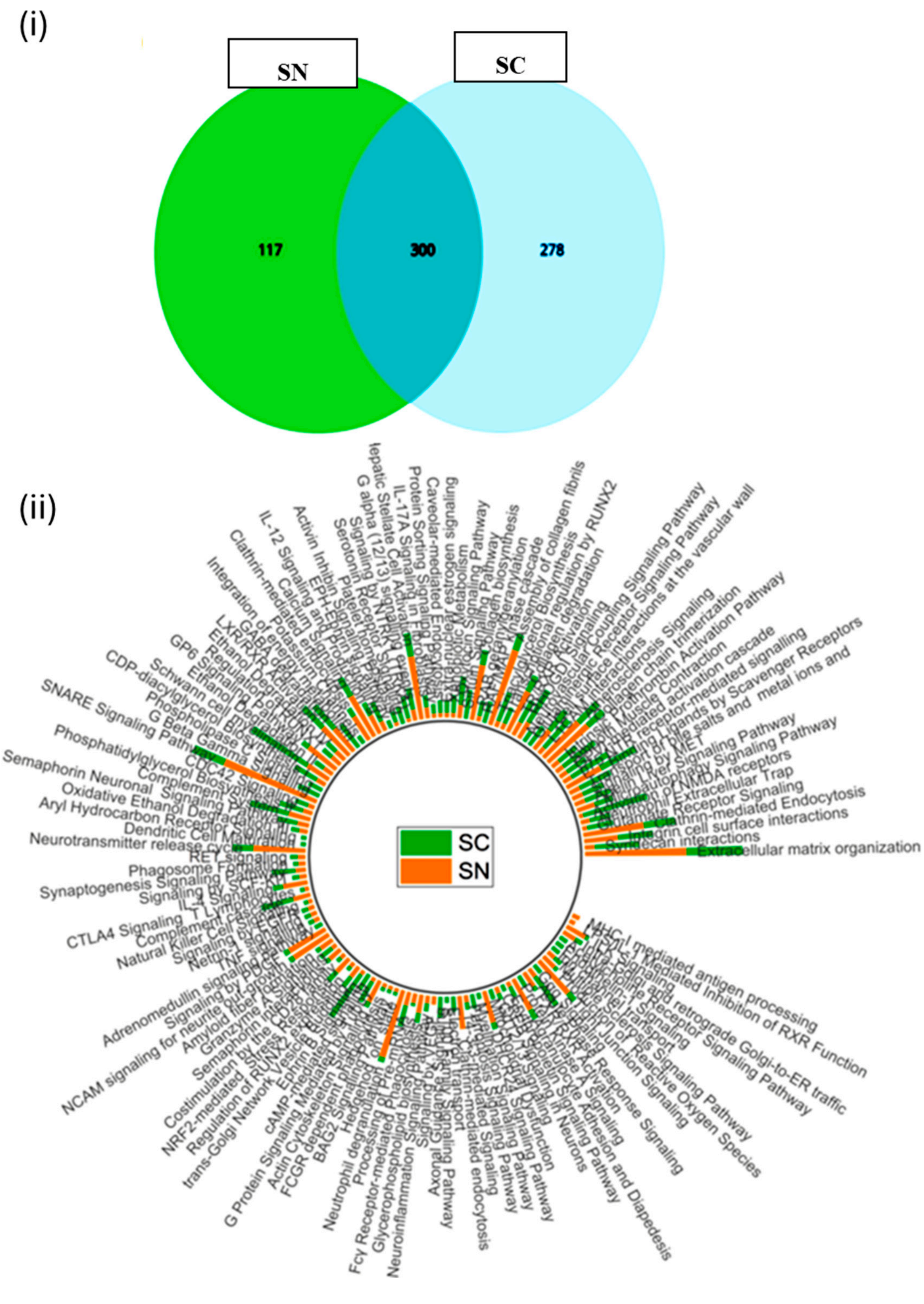

Figure S8: (i) Venn diagram representation of the canonical pathways between the SN and SC post-CCI injury. (ii) The Comparative canonical pathways analysis between the SN and SC identified by IPA. The height of the bar of individual pathways is equivalent to the significance of the identified pathways (i.e., -Log (p-value); measured using the Fisher Exact test) in relation to the IPA reference canonical pathway.

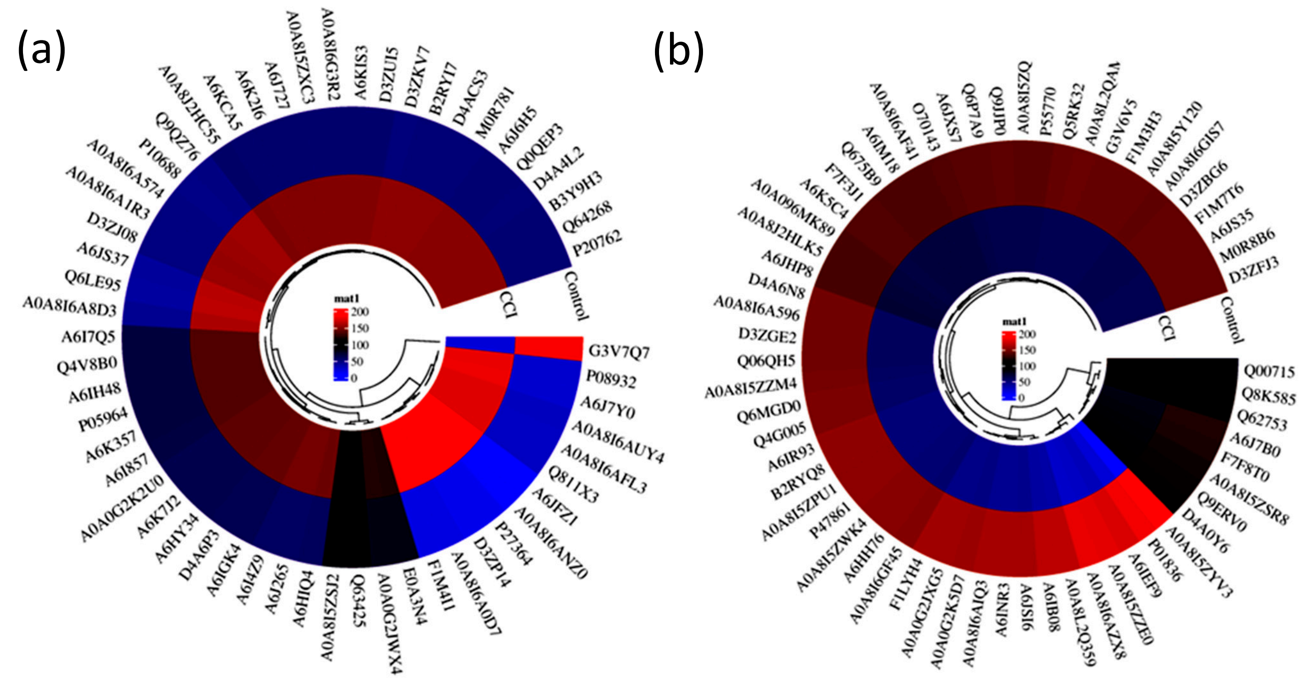

Figure S9: The abundances of the upregulated (C) and downregulated (D) proteins in the OFC of control and CCI rats along with their Uniprot Accession ID.

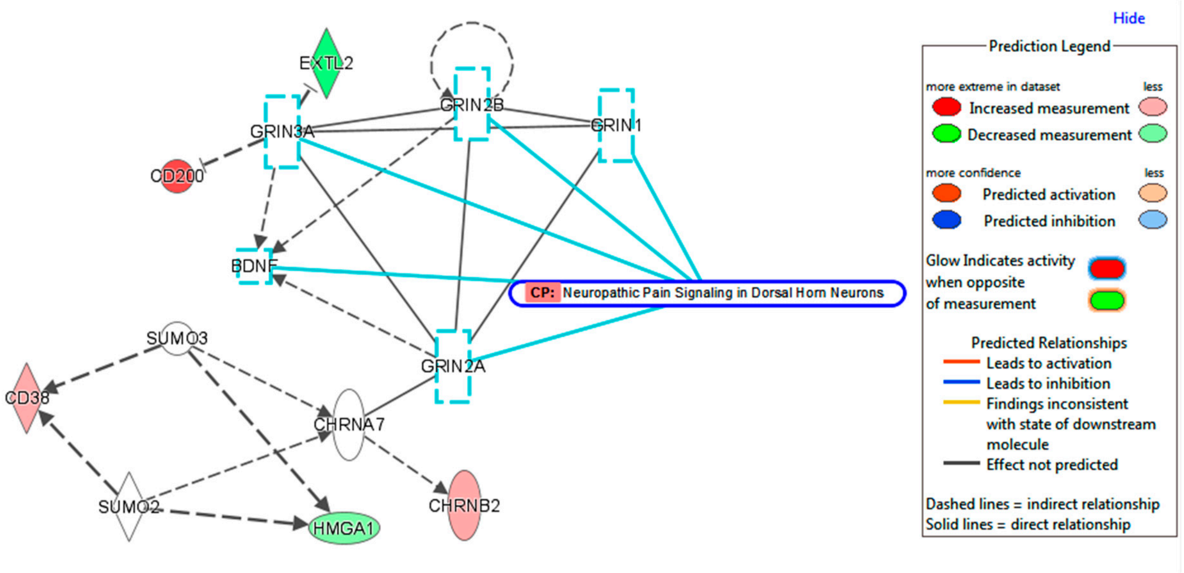

Figure S10: Relationship between specific DEPs in the OFC and canonical pathway (neuropathic pain) based on the top network predicted by IPA. Brain-derived neurotrophic factor (BDNF), CD200 molecule, CD388 molecule, cholinergic receptor nicotinic alpha and beta subunit (CHRNA7 and CHRNB2), exostosin like glycosyltransferase 2 (EXTL2), and glutamate ionotrophic receptor (GRIN1, GRIN2A, GRIN2B, and GRIN3A). Red and green nodes indicate upregulated and downregulated proteins in the dataset, respectively. Brown and blue nodes indicate predicted activation ad inhibition of proteins, respectivelyby IPA.Solid lines indicate direct connections, while dotted lines indicate indirect connections (circular arrows mean influence itself). The pointed and blunt arrow heads represent activating and inhibitory relationships, respectively. Orange lines indicate activation, blue inhibition, yellow indicates that findings are inconsistent with the state of downstream molecule, and gray indicates no predicted effect.

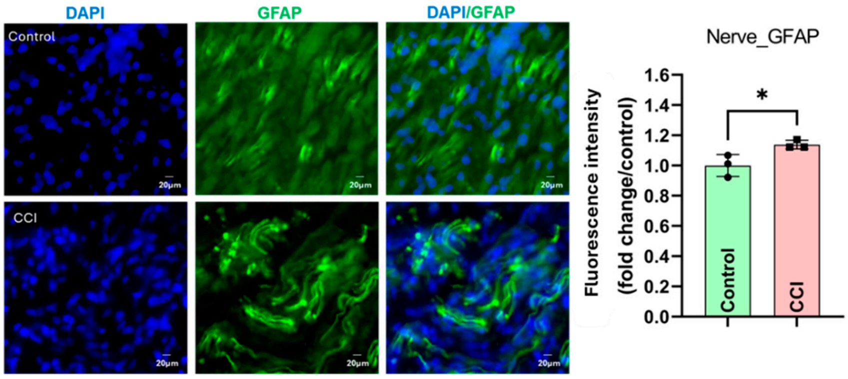

Figure S11: CCI-induced neuroinflammation in the sciatic nerve is associated with increased GFAP expression. Scale bar was set at 20 µM with 40X magnification. All values are in mean ± SD (*n*=three rats/ group). To determine statistical significance, we employed two-tailed Student's t-tests, with p-values of *p<0.05 indicating the levels of statistical significance.

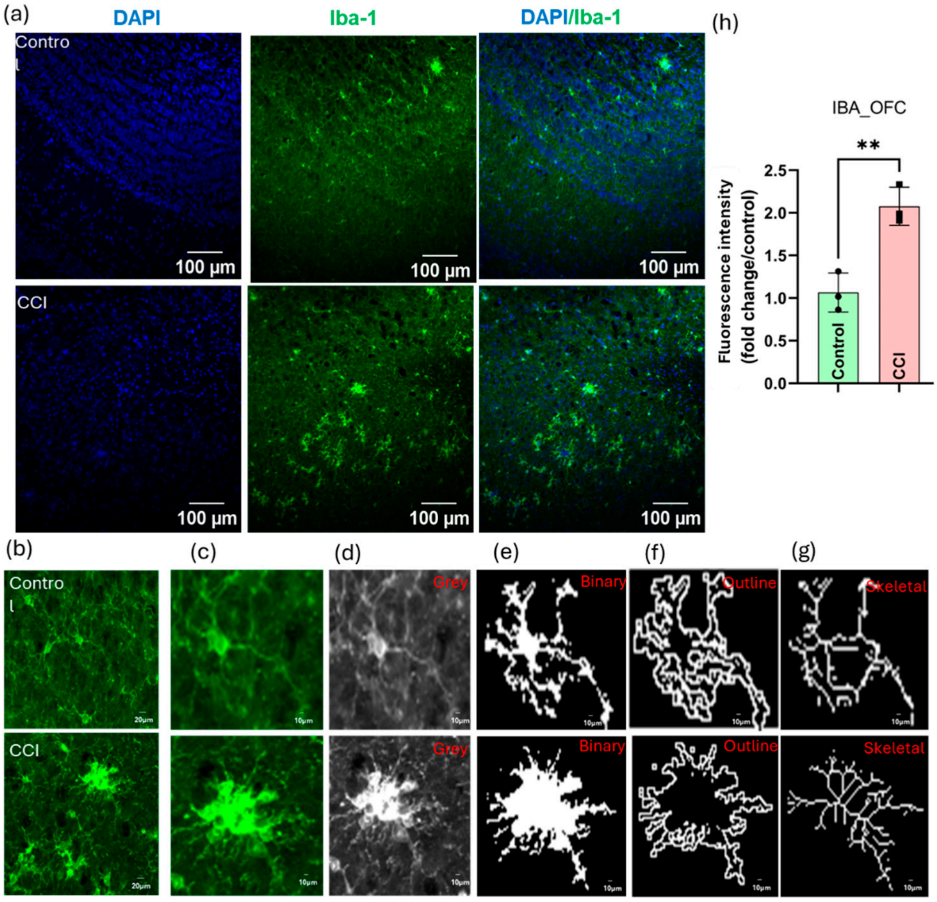

\

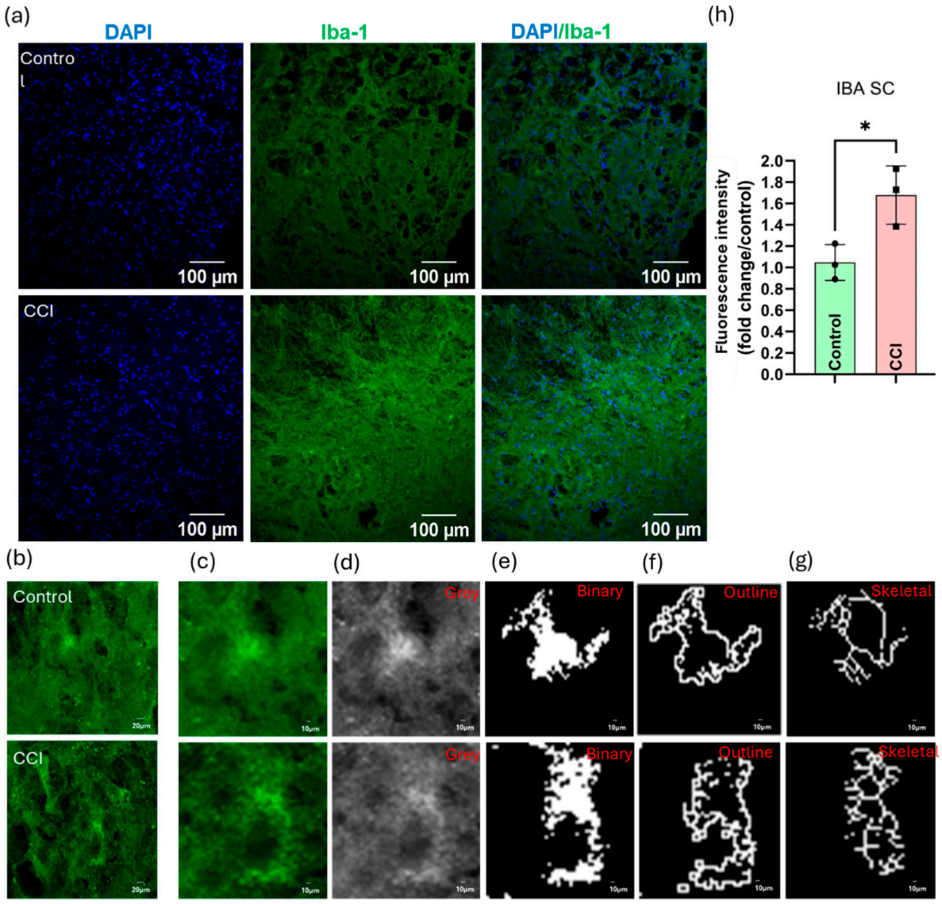

Figure S12: Microglial activation in the orbitofrontal cortex (OFC) and spinal cord (SC) following CCI. *(i)* Microglial activation in the OFC. *(a)* Representative immunofluorescence micrographs from the ipsilateral OFC showing DAPI (blue), Iba-1 (green), and merged channels in control and CCI group. Scale bar: 100 µm. *(b, c)* High-magnification microglial images (20 and 10 µm scale bars respectively) followed by *(d)* grayscale conversion, *(e)* binary, *(f)* outline tracing, and *(g)* skeletonizationused for morphological quantification of microglial ramification. *(ii)* Microglial activation in the SC post-CCI. *(a)* Representative micrographs from the ipsilateral dorsal horn of the spinal cord, specifically laminae I–IIIshowing DAPI (blue), Iba-1 (green), and merged channels.Scale bar: 100 µm. *(b, c)* High-magnification microglial morphology (20 and 10 µm scale bars respectively) analysis from ipsilateral spinal cord, including*(d)* greyscale,*(e)* binary, *(f)* outline, and *(g)* skeleton representation.*(h)* Bar graphs (right side of panels) represent quantification of Iba-1 intensity. All values are in mean ± SD (*n*=three rats/ group). To determine statistical significance, we employed two-tailed Student's t-tests, with p-values of *p<0.05 indicating the levels of statistical significance.

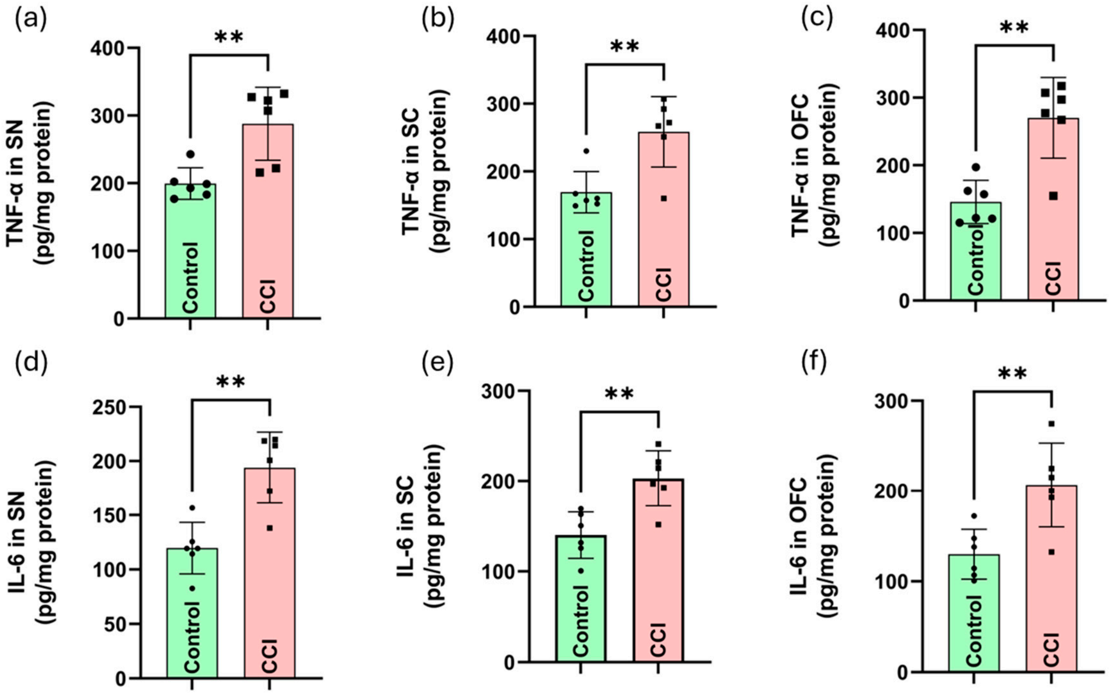

Figure S13: Effect of CCI on neuroinflammation (TNF-α and IL-6) in the sciatic nerve (a and d respectively), spinal cord (b and e respectively), and orbitofrontal cortex (c and f respectively). All values are in mean ± SD (*n*=six rats/ group). To determine statistical significance, we employed two-tailed Student's t-tests, with p-values of $^{**}p<0.05$ indicating the levels of statistical significance.

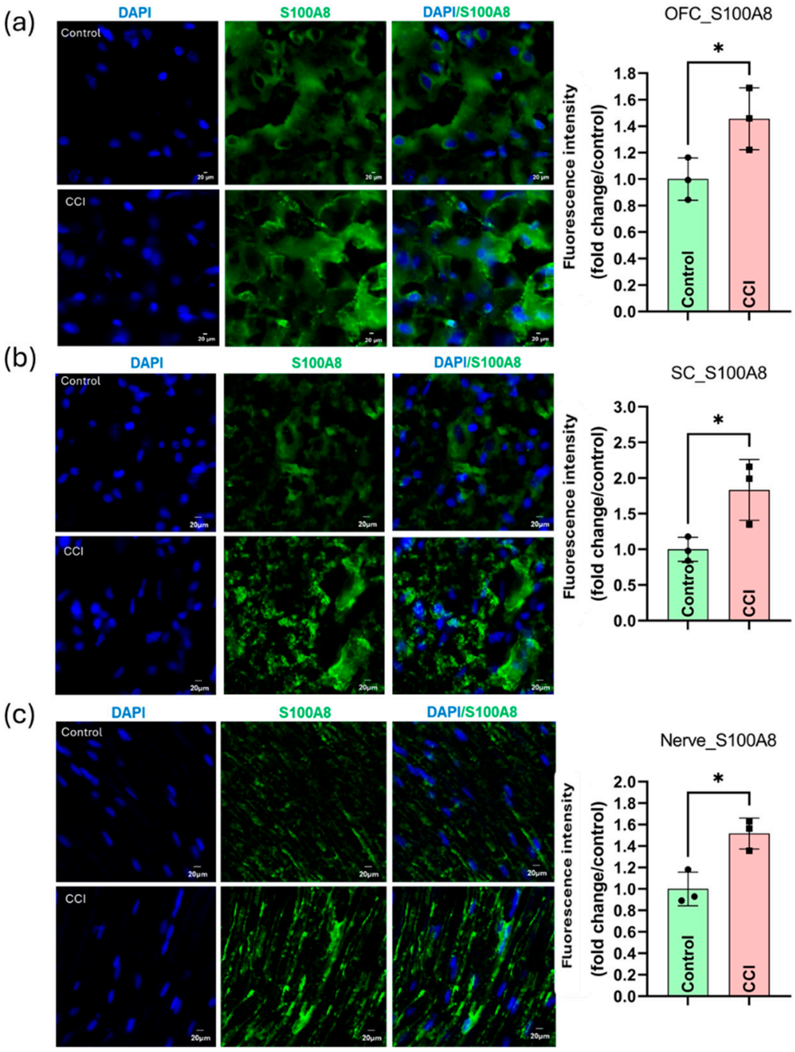

Figure S14: CCI-induced sciatic nerve injury leads to elevated S100A8 expression (red arrow) in OFC *(a)*, ipsilateral dorsal form of the SC, specifically laminae I–III *(b)* and ipsilateral sciatic nerve *(c)*, indicating peripheral and central neuroinflammation. Scale bar was set at 20 µM with 40X magnification. All values are in mean ± SD (*n*=three rats/ group). To determine statistical significance, we employed two-tailed Student's t-tests, with p-values of *p<0.05 indicating the levels of statistical significance.

Table S1: All the networks of the up and down-regulated proteins expressed in the sciatic nerve according to IPA

	Molecules in Network with Accession ID	Score	Focus mol.	Top diseases & Functions
1	Akap9, AMPH, BDNF, CASP3, CDH10, CDK5, CDK5R, CNKSR2, CRYAB, DCN, DDIT3, DLG2, DLG4, DNM1, GPNMB, GRIN1, GRIN3A, GSK3B, HMOX1, KCNA2, KCNA4, KIF5B, LGI1, MAPT, NDUFS4, PRKAR2A, PRKCE, PTEN, RB1, SH3GL2, SNAP91, SNCA, STXBP1, SYNJ1, TSC	13	12	Metabolic disease, Nervous system Development and Function, Neurological Disease
2	CKAP5, HNRNPA2B1, Mbp	3	2	Cellular Assembly and Organization, Molecular Transport, RNA Trafficking
3	CAV2, CD59, SUMO2, SUMO3	3	2	Cell Cycle, Cellular Development, Post-Translational Modification
4	POMC, SLC17A7	1	1	Endocrine System Development and Function, Lipid Metabolism, Molecular Transport
5	COL5A1, COL5A3	1	1	Cancer, Connective Tissue Disorders, Dermatological Diseases and Conditions
6	PLEC, VIM	1	1	Cellular Assembly and Organization, Cellular Function and Maintenance, Embryonic Development
7	NEFH, PIN1	1	1	Cell Morphology, Cellular Assembly and Organization, Cellular Compromise
8	E2F1, PPM1G	1	1	Cardiac Dysfunction, Cardiovascular Disease, Cellular Growth and Proliferation
9	CSF3, Tpm1	1	1	Cell Morphology, Cellular Assembly and Organization, Embryonic Development
10	DCTN2, DCTN4	1	1	Cell Morphology, Hematological System Development and Function, Inflammatory Response
11	FBXO2, GRIN1	1	1	Cell-To-Cell Signaling and Interaction, Cellular Assembly and Organization, Nervous System Development and Function
12	FADD, FAS	1	1	Neurological Disease, Organismal Injury and Abnormalities, Psychological Disorders
13	DPP10, KCND3, KCNIP1	1	1	Neurological Disease, Organismal Injury and Abnormalities, Skeletal and Muscular Disorders
14	DPYSL2, GRB2, KLC2	1	1	Hereditary Disorder, Neurological Disease, Ophthalmic Disease
15	CNTF, FGF2, FGFR1, STAT3	1	1	Cell Death and Survival, Cellular Development, Nervous System Development and Function
16	CACNA1A, MEF2A, SCN1B, SNAP25, SYT1, SYT2, SYT4, VAMP2	1	1	Cellular Assembly and Organization, Cellular Function and Maintenance, Cellular Movement

Table S2: All the networks of the up and down-regulated proteins in the spinal cord according to IPA

	Molecules in Network with Accession ID	Score	Focus mol.	Top diseases & Functions
1	BDNF, CAV1, CAV2, CHRNA7, CNKSR2, CSF3, GABRG2, GNG7, GRIN1, GRIN2A, GRIN2B, GRIN3A, KCNH5, MAP2, NECAB2, RNASE4, SLC1A1, SLITRK1, SRC, SUMO2, SUMO3, TNC	13	9	Neurological Disease, Organismal Injury and Abnormalities, Psychological Disorders
2	ITGA1, ITGB1	2	1	Cell-To-Cell Signaling and Interaction, Cellular Movement, Skeletal and Muscular System Development and Function
3	MYBL2, RBL2	2	1	Cell Cycle, Cellular Development, Connective Tissue Development and Function
4	LRRTM4, PTPRS	2	1	Cell-To-Cell Signaling and Interaction, Cellular Assembly and Organization, Cellular Function and Maintenance
5	S100A11, TSC2	2	1	Cancer, Cardiovascular Disease, Cell Morphology
6	KCNAB2, PRKAA2, PRKCZ, SQSTM1	1	1	Cell Death and Survival, Cell Morphology, Cellular Function and Maintenance
7	JUN, JUND, NQO1, RTN4, RTN4IP1, RTN4R, TRPV1	1	1	Dermatological Diseases and Conditions, Neurological Disease, Organismal Injury and Abnormalities
8	ADORA1, AP2A2, CACNG7, DLG1, GRIA1, GRIA2, GRIA3, GRIA4, GRIP1, mir-181, NR3C1, NSF, PICK1, TNF	1	1	Cell-To-Cell Signaling and Interaction, Cellular Assembly and Organization, Nervous System Development and Function

Table S3: The upregulated and downregulated proteins (with their Accession ID) expressed in the orbitofrontal cortex (OFC) post-CCI in rats

Accession ID	Protein description
(A) Upregulated proteins	
A6JFZ1	Uncharacterized protein
A0A8I6ANZ0	RAD21 homolog (S. pombe)
P27364	NADPH-dependent 3-keto-steroid reductase Hsd3b5
A0A8I6A0D7	Cd200 molecule
A0A8I6AFL3	protein-tyrosine-phosphatase
Q811X3	Carbonic anhydrase 11
A0A8I6AUY4	Mitochondrial ribosomal protein S30
P08932	T-kininogen 2
Q6LE95	Kininogen
G3V7Q7	IQ motif containing GTPase activating protein 1
F1M4I1	Catenin alpha
A0A8I6A8D3	Periplakin
P10688	1-phosphatidylinositol 4,5-bisphosphate phosphodiesterase delta-1
A0A8I6A574	NIMA-related kinase 4
D3ZJ08	Histone H3
D3ZKV7	Trinucleotide repeat containing 18
A6JS37	RCG36716, isoform CRA_e
D4ACS3	Mab-21 domain containing 2
M0R781	Dipeptidyl peptidase 9
B2RYI7	Wdr41 protein
D4A4L2	Coiled-coil domain containing 97
Q64268	Heparin cofactor 2
A0A8I6A1R3	Retinol saturase
A6J6H5	Cholinergic receptor, nicotinic, beta polypeptide 2 (Neuronal)
Q0QEP3	ATP synthase subunit beta (Fragment)
A0A8I5ZXC3	ADP-ribosyl cyclase/cyclic ADP-ribose hydrolase
A0A8I6G3R2	E3 ubiquitin-protein ligase CBL
A6KIS3	Chloride channel 3, isoform CRA_b
D3ZUI5	Protein cordon-bleu
A6KCA5	ADP-ribosylation factor 3
A6J7Y0	Orosomucoid 1
A6K2I6	RCG49687, isoform CRA_g
A6J727	Distrobrevin binding protein 1, isoform CRA_c
Q9QZ76	Myoglobin
A6J265	Tyrosylprotein sulfotransferase 2
A6IH48	RCG41300
A6HIQ4	F-box and leucine-rich repeat protein 20, isoform CRA_c
P20762	Ig gamma-2C chain C region
A0A8J2HC55	Similar to Rbl1: Retinoblastoma-like protein 1 (Rattus norvegicus)
Q63425	Periaxin
A6IGK4	TBC1 domain family, member 15
D4A6P3	Shootin-1

A6HY34	Similar to ovary-specific MOB-like protein (Predicted), isoform CRA_b
A6I4Z9	Synaptic vesicle glycoprotein 2c
A6K7J2	Hypothetical LOC315216 (Predicted), isoform CRA_b
A0A0G2K2U0	Phosphatidylserine synthase
A0A8I5ZSJ2	Poly(A) binding protein, cytoplasmic 1-like 2A
E0A3N4	Serpina3n-like protein
A0A0G2JWX4	Keratin 2
A6I857	RCG39957, isoform CRA_b
B3Y9H3	S100 calcium binding protein A10
Q4V8B0	Oxidation resistance protein 1 3
A6I7Q5	Olfactory receptor 223 (Predicted)
A6K357	RCG51861
D3ZP14	Carboxylic ester hydrolase
P05964	Protein S100-A6
B) Down-regulated proteins	
A0A8J2HLK5	Similar to Otoferlin
Q675B9	Taste receptor type 2 member
A0A8I6AF41	Triokinase/FMN cyclase
A6IM18	Similar to ovostatin-2
F1M3H3	Fraser extracellular matrix complex subunit 1
A6K5C4	RCG24416, isoform CRA_a
A0A8I5ZYV3	Janus kinase and microtubule interacting protein 1
O70143	SHC-transforming protein 3
Q9ERV0	Pkd1 (Fragment)
Q9JJP0	Sodium-dependent phosphate transporter 1
F7F3J1	Myl9 protein
A6JHP8	Similar to RIKEN cDNA 2810048G17 gene
A0A8I5ZZE0	Bromodomain and WD repeat domain containing 3
Q8K585	High mobility group protein HMG-I/HMG-Y
A0A8I6GIS7	RAB11 binding and LisH domain, coiled-coil and HEAT repeat containing
A6JXS7	RCG32197, isoform CRA_a
Q6P7A9	Lysosomal alpha-glucosidase
A0A8I5ZSR8	Caseinolytic mitochondrial matrix peptidase chaperone subunit B
A0A8I5ZQ74	Similar to Histidine triad nucleotide-binding protein 1 (Adenosine 5-monophosphoramidase) (Protein kinase C inhibitor 1) (Protein kinase C-interacting protein 1) (PKCI-1)
P55770	NHP2-like protein 1
A0A8L2QAM9	ADP-ribosylation factor related protein 1
A0A0G2K5D7	Sperm antigen with calponin homology and coiled-coil domains 1
D3ZBG6	aminoacyl-tRNA hydrolase
F1M7T6	Translocon-associated protein subunit gamma
A6JS35	RCG36716, isoform CRA_a
M0R8B6	Tubulin beta chain
A6J7B0	Desmoplakin, isoform CRA_b
A0A8I5Y120	BRCA1 associated protein
Q5RK32	RGD1308009 protein (Fragment)
P47861	Synaptotagmin-5
G3V6V5	Sodium/potassium-transporting ATPase subunit beta
A0A8I6A596	Mitotic arrest deficient 1 like 1

A0A096MK89	Adhesion G protein-coupled receptor V1
Q06QH5	NADH-ubiquinone oxidoreductase chain 2
D4A0Y6	Marker of proliferation Ki-67
A0A8L2Q359	2-iminobutanoate/2-iminopropanoate deaminase
Q62753	Syntaxin-binding protein 2
D4A6N8	T-box brain transcription factor 1
D3ZGE2	Myeloperoxidase
A0A8I5ZZM4	Testis expressed 30
Q00715	Histone H2B type 1
Q4G005	General transcription and DNA repair factor IIH helicase subunit XPB
B2RYQ8	Large subunit ribosomal protein L36a, isoform CRA_a
A0A0G2JXG5	Golgi associated PDZ and coiled-coil motif containing
Q6MGD0	Protein CutA
A0A8I6GF45	E3 ubiquitin-protein ligase listerin
A6IB08	Similar to Rho-GTPase-activating protein 25 (Predicted)
F7F8T0	Exostosin-like glycosyltransferase 2
A6HH76	RCG35210
A0A8I5ZPU1	Chromosome 10 open reading frame, human C5orf58
A6IR93	RAB, member of RAS oncogene family-like 3 (Predicted), isoform CRA_b
D3ZFJ3	SH3 domain-binding protein 1
F1LYH4	FYN binding protein 2
A6JSI6	Reticulon 4 receptor
A0A8I5ZWK4	Glyceraldehyde 3-phosphate dehydrogenase catalytic domain-containing protein
A6INR3	Four and a half LIM domains 2, isoform CRA_a
A0A8I6AIQ3	Family with sequence similarity 98, member C
A6IEF9	Zinc finger, C3HC-type 1 (Predicted)
A0A8I6AZX8	Leucine rich repeat containing 75A
P01836	Ig kappa chain C region, A allele

Table S4: **All the networks of the up and down-regulated proteins in the OFC according to IPA**

	Molecules in Network with Accession ID	Score	Focus mol.	Top diseases & Functions
1	BDNF, CD200, CD38, CHRNA7, CHRNB2, EXTL2, GRIN1, GRIN2A, GRIN2B, GRIN3A	8	5	Neurological disease, organismal injury and abnormalities, skeletal and muscle disorders
2	DRD1, S100A10	2	1	Behavior, endocrine system disorders, gastrointestinal disease
3	GABBR2, JAKMIP1			Development disorder, hereditary disorder, neurological disease
4	S100A6, TSC2	2	1	Cellular development, cellular growth and proliferation, connective tissue development and function
5	Clip1, IQGAP1. MTOR	2	1	Cell morphology, nervous system development and function, tissue morphology
6	APP, MMP16, RTN4, RTN4R	2	1	Cell morphology, cellular compromise, cellular development
7	Dmd, DRD2, DTNB DTNBP1, UTRN	1	1	Cell-to-cell signaling and interaction, drug metabolism, nervous system development and function
